# Lignin and Nanolignin: Next-Generation Sustainable Materials for Water Treatment

**DOI:** 10.1021/acsabm.4c01563

**Published:** 2025-02-11

**Authors:** Camilla H. M. Camargos, Liu Yang, Jennifer C. Jackson, Isabella C. Tanganini, Kelly R. Francisco, Sandra R. Ceccato-Antonini, Camila A. Rezende, Andreia F. Faria

**Affiliations:** † Departamento de Artes Plásticas, Escola de Belas Artes, Universidade Federal de Minas Gerais, Belo Horizonte, Minas Gerais 31270-901, Brazil; ‡ Engineering School of Sustainable Infrastructure and Environment, Department of Environmental Engineering Sciences, University of Florida, Gainesville, Florida 32611-6540, United States; § Departamento de Tecnologia Agroindustrial e Socioeconomia Rural, Universidade Federal de São Carlos, Araras, São Paulo 13600-970, Brazil; ∥ Departamento de Ciências da Natureza, Matemática e Educação, Universidade Federal de São Carlos, Araras, São Paulo 13600-970, Brazil; ⊥ Departamento de Físico-Química, Instituto de Química, Universidade Estadual de Campinas, Campinas, São Paulo 13083-970, Brazil

**Keywords:** lignocellulose, lignin nanoparticles, drinking water, adsorption, flocculation, photocatalysis, membrane filtration, antimicrobial agents

## Abstract

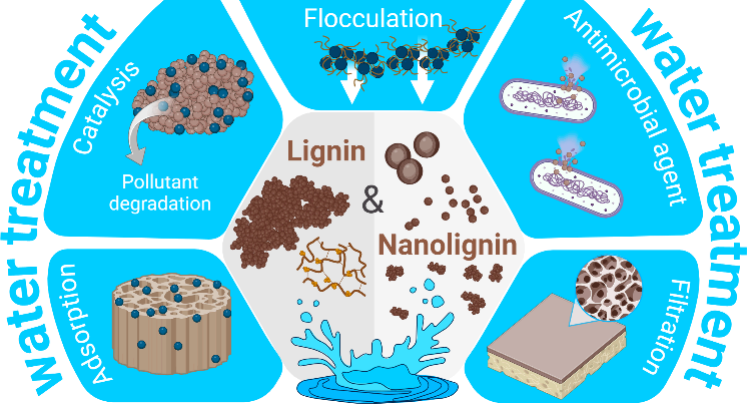

Water scarcity, contamination, and lack of sanitation are global issues that require innovations in chemistry, engineering, and materials science. To tackle the challenge of providing high-quality drinking water for a growing population, we need to develop high-performance and multifunctional materials to treat water on both small and large scales. As modern society and science prioritize more sustainable engineering practices, water treatment processes will need to use materials produced from sustainable resources via green chemical routes, combining multiple advanced properties such as high surface area and great affinity for contaminants. Lignin, one of the major components of plants and an abundant byproduct of the cellulose and bioethanol industries, offers a cost-effective and scalable platform for developing such materials, with a wide range of physicochemical properties that can be tailored to improve their performance for target water treatment applications. This review aims to bridge the current gap in the literature by exploring the use of lignin, both as solid bulk or solubilized macromolecules and nanolignin as multifunctional (nano)materials for sustainable water treatment processes. We address the application of lignin-based macro-, micro-, and nanostructured materials in adsorption, catalysis, flocculation, membrane filtration processes, and antimicrobial coatings and composites. Throughout the exploration of recent progress and trends in this field, we emphasize the importance of integrating principles of green chemistry and materials sustainability to advance sustainable water treatment technologies.

## 1 Introduction

Water resource sustainability remains one of the most significant challenges of the 21st century because many communities worldwide lack access to safe water and sanitation. According to indicators of the United Nations Sustainable Development Goal 6 (SDG 6: ensure access to water and sanitation for all), updated in February 2023, two billion people, 26% of the world population, were deprived of safely managed drinking water services in 2020.^1^ Additionally, less than 60% of household wastewater was safely treated in that same year. Pollution and climate change further exacerbate the problem of supplying clean water to a growing population in the coming decades.

It is crucial to ensure that there are sufficient supplies of clean and drinkable water to support human life, promote socioeconomic development, and protect the environment. To meet this pressing demand while minimizing stresses on established water systems, current water treatment strategies need to be enhanced and supplemented with water from nontraditional sources. Options to increase water supplies include improving drinking water and wastewater treatments,^2^ as well as reusing or recycling water.^3^

Wastewater treatment plants play a critical role in mitigating the environmental impacts associated with the discharge of untreated effluents into natural water systems. They enable the remediation of wastewater and its reuse for nonpotable applications, such as agricultural irrigation and as a cooling source for industrial processes.^4^ Conversely, drinking water treatment plants or point-of-use devices employ various sequential technologies, such as physical separation filters, carbon adsorbents, ion-exchange resins, reverse osmosis (RO) membranes, and ultraviolet (UV)-light disinfection.^5^ In this context, a variety of biological, organic, and inorganic water contaminants can be removed using multiple strategies, including physical methods like adsorption, coagulation/flocculation, and membrane filtration, as well as chemical methods like catalysis and advanced oxidation approaches.^6^ Although effective, many conventional treatment methods are often limited to laboratory-scale studies or specific tasks,^2,6,7^ because they entail high capital and operational costs. These expenses arise from the necessity of specialized gray infrastructure, the use of costly nonreusable or nonrecyclable materials, energy-intensive processes, and the challenges associated with the safe disposal of sludges and spent materials, such as adsorbents.^8^ Additionally, problems like the fouling of commercial water purification membranes further drive up maintenance and replacement costs.^9,10^

To make these water treatment approaches feasible for pilot- or large-scale applications, there is a need to develop cost-effective, nontoxic, highly efficient, and renewable/reusable platforms. In this context, the use of plant- and waste-derived materials, such as biopolymers and biomacromolecules such as cellulose and lignin, emerges as a promising alternative to traditional water remediation technologies. These biomaterials, extracted from lignocellulosic biomasses, have been explored as sorbents,^11^ flocculants,^12^ as well as functionalized membranes,^13,14^ antimicrobial agents,^15,16^ and catalysts.^17,18^

Biomass-derived materials, particularly lignin, are frequently extracted without regard to their intended use or disposal. However, developing value-added applications beyond energy production through combustion can create new revenue streams and reduce waste accumulation. That said, the extraction and processing of biomaterials often require intensive chemical treatments, which can generate toxic byproducts and elevate both operational and capital costs. Adopting methods like soda extraction, which is low cost and has minimal environmental impact,^19^ along with transitioning to greener, recyclable alternatives, such as deep eutectic solvents,^20^ shows promise for fostering more sustainable and economically viable life cycles. This approach could be particularly beneficial in the production of lignin-based water treatment platforms.

### 1.1 Extraction of Bulk Lignin and Preparation of Nanostructured Lignin-Based Materials

**Figure 1:**
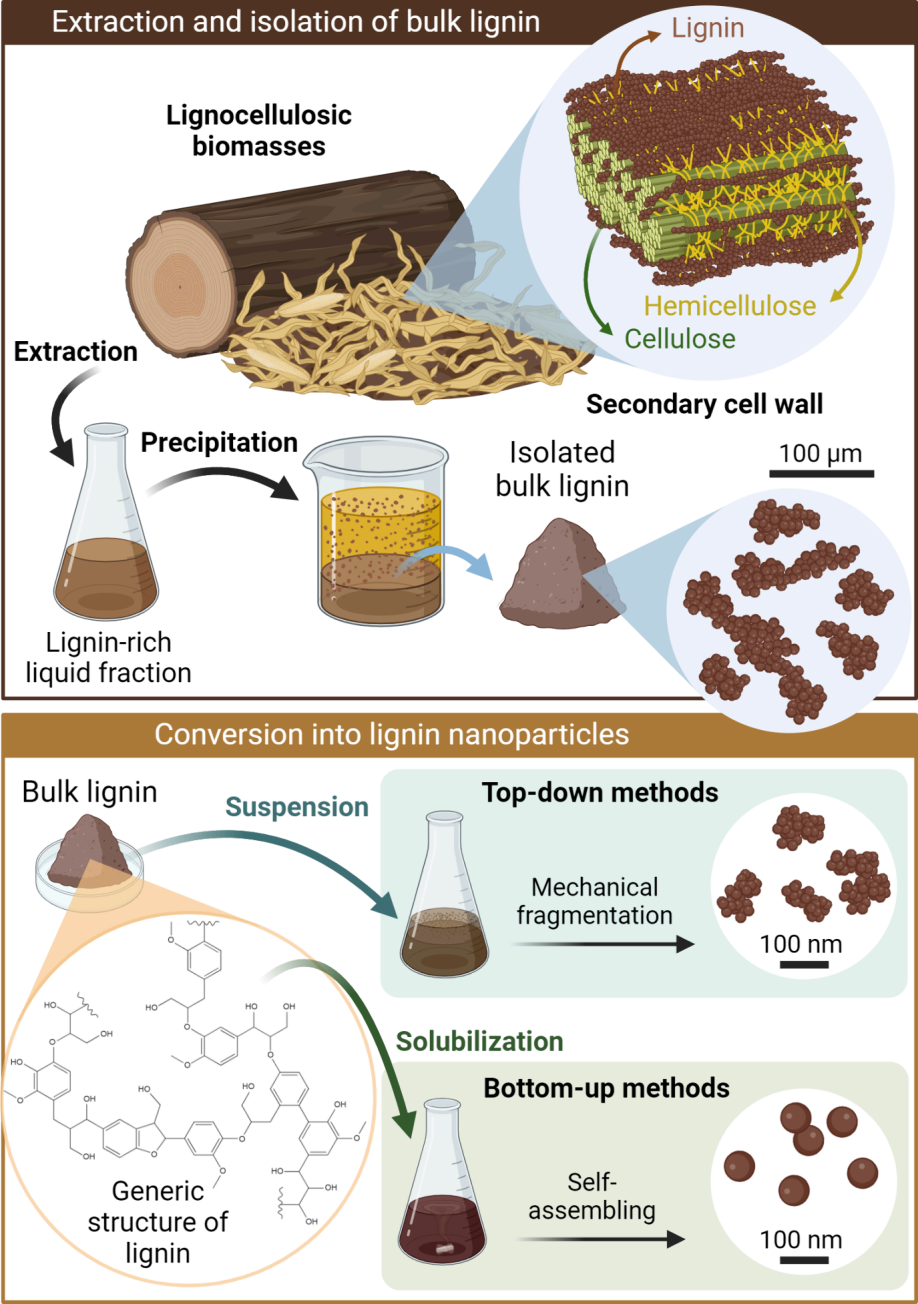
Depiction of the structure of the secondary cell wall in lignocellulosic biomasses, such as wood and crop residues, and a generic rationale for the extraction of lignin into a lignin-rich liquid fraction followed by the precipitation and isolation of bulk lignin. Afterward, bulk lignin can be converted into lignin nanoparticles through top-down approaches, which involve the mechanical fragmentation of lignin aggregates in suspension, or bottom-up approaches, which involve the solubilization of lignin macromolecules and their self-assembly into nanospheres. Created in BioRender. Camargos, C. (2025) https://BioRender.com/r61b405

Lignocellulosic biomasses comprise complex cell wall structures derived from different plant tissues.^21^ The primary cell wall is a thin, hydrated, and flexible structural layer that surrounds growing plant cells.^22^ Once growth stops, a thickened secondary cell wall forms inside the primary cell wall, mainly in cells responsible for defense, support, and water and nutrient transport, such as fibers in the xylem tissue.^23^ As depicted in Figure 1, the secondary cell wall is predominantly composed of bundles of highly ordered cellulose microfibrils surrounded by hemicelluloses and embedded into a highly cross-linked three-dimensional lignin matrix.^24^

**Figure 2:**
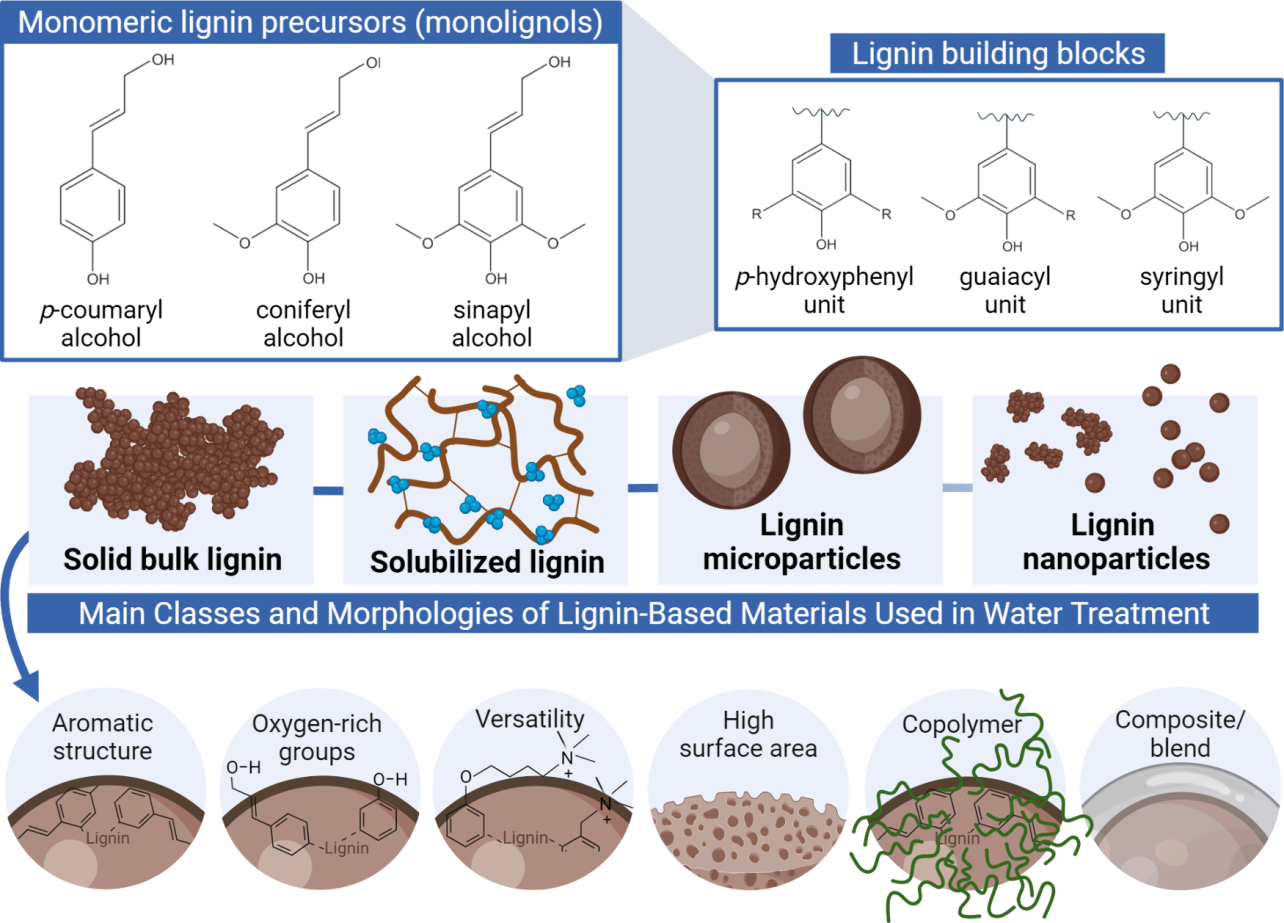
Chemical structure of the primary monolignols, which are the precursors of lignin, and the fundamental structural units (building blocks) of lignin macromolecules. Schematic representation of lignin-based functional macro-, micro-, and nanomaterials, as well as fundamental properties related to the application of solid bulk lignin, solubilized lignin, lignin microparticles, and lignin nanoparticles for water treatment processes. Created in BioRender. Camargos, C. (2025) https://BioRender.com/v92u223.

Lignin accounts for 15–40% of the dry weight of most terrestrial plants,^25^ including gymnosperms (softwoods) and angiosperms (hardwoods and grasses). The structural differences in lignins among various plant categories primarily stem from the proportions of their repeating units (Figure 2). Softwoods are predominantly composed of guaiacyl units with only minor amounts of *p*-hydroxyphenyl units. In contrast, hardwoods contain both guaiacyl and syringyl units, while grasses typically include all three types of units, often exhibiting slightly elevated levels of *p*-hydroxyphenyl.^26^ Moreover, the molecular weight of native lignin can vary depending on the plant source and extraction method, ranging from 7000–8000 g/mol in grasses^27^ to 78400 g/mol in softwoods.^25^

Beyond providing water-conducting properties and mechanical reinforcement,^9^ native lignin plays a crucial role in maintaining the integrity of lignified extracellular structures against biological, chemical, and physical decay.^28^ Particularly in the papermaking and bioethanol industries, this aromatic biomacromolecule can be extracted as lignin-rich liquors (liquid fractions) using physical/mechanical, chemical, and enzymatic methods.

After lignocellulosic biomass undergoes milling steps, lignin extraction is carried out using diverse strategies such as acid hydrolysis, alkaline treatment, Kraft or sulfite pulping, organosolv processes, deep eutectic solvent methods, and enzymatic hydrolysis.^29^ These extraction processes often involve oxidation, which results in the formation of oxidized functional groups, such as aldehydes, carboxylic acids, and quinones.^30^ Typically, acid hydrolysis pretreatments are not particularly efficient to enable lignin removal, but a preliminary acid step acts in hydrolyzing hemicellulose from lignin–carbohydrate complexes,^19,31^ thereby facilitating the isolation of lignin-rich fractions in subsequent stages. Below are the most common extraction methods along with the primary properties of extracted lignins:

- Alkaline treatments: Biomass is processed using alkaline solutions, such as sodium, ammonium, potassium, and calcium hydroxides with concentrations ranging from 0.5% to 30%.^32^ This process occurs under varying conditions of temperature (25–180 °C), pressure (1–17 atm), time (5–60 min), and solid-to-liquid ratios (1:10–1:2).^32^ The lignins extracted through alkaline methods, including soda lignin, have a high phenolic content due to the cleavage of aryl ether bonds.^30,33,34^ They also exhibit a moderate-to-high content of negatively charged ionizable surface groups, ranging from 0.16 to 2.0 mmol/g,^27,35^ and molecular weights (*M*_n_) between 1000 and 6000 mol/g.^27,35^ While alkaline lignin (AL) is highly soluble in alkaline solutions, its solubility decreases under neutral or acidic pH because of reduced ionization of the functional groups.
- Kraft pulping: Biomass is processed using high concentrations of sodium hydroxide and sodium sulfide, with effective alkali levels ranging from 12% to 24% and sulfidity of up to 30–50%,^36^ at elevated temperatures exceeding 160 °C.^37^ This process yields significant cleavage of β-O-4 and α-O-4 linkages, generating a substantial amount of nonetherified phenolic hydroxyl groups and leading to considerable condensation of polyphenylpropene units.^34^ Kraft lignin (KL) contains 1–3% sulfur^38^ and is predominantly hydrophobic, displaying limited solubility in both water and organic solvents. However, similar to AL, its solubility improves significantly at alkaline pH due to the deprotonation of phenolic and carboxylic groups. The molecular weight of KL varies considerably from 1000 to 15000 mol/g.^30,34^
- Sulfite pulping: This process utilizes solutions that contain sulfur dioxide and hydrogen sulfite ions at elevated temperatures, which results in the production of lignosulfonates (LS). These water-soluble anionic polyelectrolytes are rich in sulfonic acid functional groups,^39−41^ exhibiting excellent binding and emulsifying properties. Additionally, they have a remarkably broad molecular weight distribution (*M*_w_, 5000–400000 mol/g).^42^
- Organosolv processes: Biomass is fractionated through solubilization in mixtures of water and organic solvents, such as ethanol and methanol, at temperatures between 100 and 250 °C, and typically in the presence of a catalyst.^38^ Organosolv lignin (OL) is devoid of sulfur and is soluble in a wide range of organic solvents,^30^ including ethanol. It possesses a high phenolic hydroxyl content, which can reach up to 4 mmol/g.^35^ With low molecular weights ranging from 500 to 5000 mol/g,^30^ OL demonstrates narrow polydispersity, high purity, and minimal carbohydrate contamination.^25^
- Deep eutectic solvents: Emerging solvents are increasingly being recognized for their effectiveness in the fractionation of lignocellulosic biomass, primarly due to their ability to selectively dissolve and extract lignin.^43^ They accomplish this by breaking the bonds between lignin and carbohydrates, as well as the β-O-4 bonds present in lignin.^38^ Lignin extracted using these ecofriendly, cost-effective, and recyclable solvents has a relatively low molecular weight (490–2600 g/mol), small polydispersity, and poor thermal stability.^38^
- Enzymatic hydrolysis: This method is commonly employed in bioethanol production and utilizes cellulose hydrolases to break down the cellulose fraction of lignocellulosic substrates. This process produces enzymatic hydrolysis lignin (EHL) as a residual solid fraction (byproduct). EHL is sulfur-free but exhibits low purity, containing up to 50% carbohydrates and protein impurities. Its minimal oxidation allows it to maintain a structure reminiscent of native lignin, in contrast to chemically recovered lignins, which tend to be more degraded. This preservation is reflected in the retention of β-O-4 bonds and a lower degree of degradation. Furthermore, EHL has a relatively high molecular weight, typically ranging from 5000 to 10000 g/mol.^38^

These methods separate different types of bulk lignin residues into liquid fractions, which can then be precipitated by adding acids or excess water (Figure 1). For instance, the spent liquors of microwave-assisted extraction in organic acid aqueous solutions^44^ or organosolv extraction^45^ are diluted with the addition of excess water to precipitate acid and organosolv lignins, respectively. Similarly, the lignin-rich aqueous fractions of alkaline treatments,^31^ enzymatic hydrolysis,^29^ and Kraft and sulfite processes^46^ undergo acidification to isolate technical lignins respectively in the form of AL, EHL, sulfonated Kraft lignin (SKL), and LS. LignoBoost lignin is also precipitated from Kraft black liquors by acidification, preferentially using carbon dioxide (CO_2_).^47^ Still, in the LignoBoost process, after an initial filtration step, the lignin slurry is acidified again, followed by another filtration step and subsequent washing. Extraction and purification methods disrupt the native 3D structure of lignin to varying extents. As a result, extracted and technical lignins consist of fragments of the original structure with varying sizes and chemical functionalities.

Subsequently, different types of isolated lignin can be converted into lignin nanoparticles (LNPs) using either top-down or bottom-up approaches (Figure 1). Top-down methodologies involve the mechanical fragmentation of large aggregates of bulk lignin into irregularly globular-shaped LNPs, with high polydispersity (average diameter of 10–450 nm),^27,48^ achieved mainly through high shear homogenization or probe sonication (cavitation). On the other hand, bottom-up methods usually involve dissolving lignin in an aqueous organic solvent, typically acetone or tetrahydrofuran (THF), and then inducing the formation of LNPs through solvent exchange or precipitation with the addition of an antisolvent, commonly water.^49^ This process drives the self-assembly of amphiphilic lignin macromolecule moieties into regular spherical colloidal lignin particles (LPs) with an average diameter below 100 nm.^27,49^ Both types of LNPs generally present a highly negative surface charge, good colloidal stability, and outstanding antioxidant properties.

### 1.2 Structure–Property Relationships Promoting the Applicability of Lignin in Water Treatment Technologies

Lignin is formed through the *in situ* enzymatic polymerization of monolignols, including *p*-coumaryl, coniferyl, and sinapyl alcohols. These monolignols primarily associate via alkyl–aryl ether bonds (β-O-4′) into the fundamental structural units or building blocks: *p*-hydroxyphenyl, guaiacyl, and syringyl,^49,50^ as shown in Figure 2. As a result, native lignin is highly heterogeneous and hydrophobic. However, chemical delignification treatments may alter and decrease this hydrophobicity by fragmenting cross-linked units, reducing molecular weight, and incorporating hydrophilic functional groups into extracted (technical) lignins.^51,52^

Lignin, whether in the form of solubilized or solid bulk materials such as oxidized, sulfomethylated, and polymerized (copolymers) or as dispersed micro- and nanoparticles (Figure 2), has been systematically regarded as a versatile, sustainable, and effective strategy in the realm of environmental engineering and water treatment and decontamination.^53−56^ This polyphenolic macromolecule is a multifunctional material that can be used not only to change the state of a plethora of pollutants, for example, moving contaminants from aqueous to the solid phase by adsorption mechanisms, but also to degrade contaminants via advanced oxidation processes, such as photocatalysis.

Figure 2 illustrates some critical structure–property relationships of lignin. The aromatic and carbon-rich nature of lignin, as well as its abundance of phenolic groups, contributes to its high photocatalytic behavior.^57^ The polyphenolic structure imparts lignin with excellent antimicrobial properties due to interactions favored with the cell wall of microorganisms.^15^ Additionally, the presence of a significant content of oxygen-rich functional groups, including methoxyl, hydroxyl (both phenolic and aliphatic), carbonyl, and carboxylic groups,^58^ renders lignin with outstanding versatility for surface chemistry modification.^27^ Such amenability for chemical functionalization enhances the interactions with various contaminants and their removal through adsorption,^59,60^ flocculation,^61^ and membrane filtration.^52^ Moreover, this characteristic also supports the use of cost-effective lignin-based copolymers, hybrids, composites, and blends. Because lignin can easily self-assemble into supramolecular structures and networks,^62,63^ the specific surface area of lignin-based materials can be tailored to improve its performance in water treatment processes.^59^

In this review, we comprehensively and systematically examine the utilization of different types of lignin in various water treatment processes, including adsorption, catalysis, flocculation, membrane filtration, and development of antimicrobial coatings and composites. The growing number of publications delving into such applications enlightens the promise of lignin-based strategies in this field, as reflected by a steady output of reviews dealing with the application of lignin in water treatment, either as focused reviews^64−72^ or as broader state-of-the-art articles covering multiple applications of lignin^69,73,74^ and other biomacromolecules.^75,76^ Previous comprehensive reviews have examined lignin-based adsorbents^64−66^ and flocculants,^73,77^ especially with regard to lignin-derived carbon materials,^69−71^ as well as lignin-derived materials and nanomaterials used as catalysts, including their use for the (photo)degradation of wastewater pollutants.^74,78,79^ Our comprehensive exploration provides an in-depth understanding of each of the five specific applications, including discussions of their mechanisms, challenges, and prospects. Moreover, we discuss key performance indicators, compare lignin-based methods with traditional approaches, and identify advancements, as well as existing gaps and perspectives. The review aims to highlight the potential of lignin as a promising material for water treatment technologies, with a specific focus on its application in small- and medium-scale strategies.

## 2 Lignin-Based Adsorbents for Water Treatment and Remediation

Lignin has been used as a widespread precursor to produce carbonaceous adsorbents such as char and activated carbon (AC). Lignin-derived biochar and AC, obtained by either pyrolysis or hydrothermal carbonization, present several advantages related to simultaneous chemisorption and physisorption mechanisms, which promote the adsorption of multiple contaminants.^80^ These carbonaceous porous materials have been used in water and wastewater treatment to adsorb chemicals like *p*-nitrophenol^81^ and 2,4-dichlorophenoxyacetic acid (2,4-D),^82^ cationic^83,84^ and anionic dyes,^85,86^ pharmaceuticals such as chloramphenicol and tetracycline,^87,88^ oils,^89^ and various inorganic pollutants, including heavy metals^90,91^ and phosphate ions.^92^

Similarly, macro-, micro-, and nanostructured noncarbonaceous lignin-based materials have been investigated as outstanding candidates to adsorb many organic and inorganic contaminants from wastewater discharges (Table 1). Some studies have shown that precipitated lignin, in both single-component and multicomponent systems, can surpass the performance and adsorption capacities of many common adsorbents, such as peat and leonardite, as well as ACs and biochars.^93^ Lignin presents a significant amount of oxygen-rich functional groups, amenability for surface chemistry functionalization,^27^ moderate-to-high specific surface area, and the ability to self-assemble into supramolecular structures and networks.^62,63^

**Table 1:** Types of Lignin-Based Adsorbents, Target Contaminants, Mechanism of Adsorption, Adsorption Efficiency, and Experimental Conditions under Which These Biosorbents Have Been Tested

lignin-based adsorbents	target contaminants	mechanisms	adsorption efficiency	experimental conditions	refs
lignin-based aromatic porous polymers (methods: corncob lignin dissolved in 1,2-dichloroethane and cross-linked with 1,4-dichloroxylene or 4,4′-bis(chloromethyl)-1,1′-biphenyl in the presence of anhydrous FeCl_3_ as a catalyst)	chemicals (bisphenol A and bisphenol AF)	π–π interactions and pore adsorption (pore filling)	maximum adsorption capacity of up to 398.59 mg/g, which was stable in the pH range 2–10 at 25 °C	batch experiments: analyzed pH range, 2–12; analyzed temperature, 25–45 °C; dose of lignin adsorbent, 0.5 g/L; contaminant concentration, 200 mg/L; surface area, up to 1144.23 m^2^/g; total pore volume, 0.4468 cm^3^/g; regeneration methodology, desorption and regeneration were performed with ethanol and filtration steps; reusability, 5 adsorption–desorption cycles	(59)
modified lignin grafted with acrylic acid or aminated lignin (methods: dried commercial lignin solubilized in methanol and functionalized with acrylic acid in the presence of free radical initiators or reacted with 1,2-dichloroethane and anhydrous aluminum chloride to produce chlorinated lignin and then subsequently reacted with *N*,*N*-dimethylformamide and ethylenediamine)	chemicals (aniline and 2,4,6-TNT)	hydrogen-bonding and electrostatic interactions	maximum adsorption capacities of 79.1 mg/g for aniline and 55.7 mg/g for TNT in neutral solution (pH 7) at 15 °C	batch experiments: analyzed pH range, 2–11; analyzed temperature, 15–55 °C; dose of lignin adsorbent, 1 g/L; contaminant concentration, 100 or 500 mg/L; surface area, 22.01 m^2^/g; total pore volume, 0.145 cm^3^/g; regeneration methodology, static washing with hydrochloric acid, acetone, or ethanol and filtration steps; reusability, 8 adsorption–desorption cycles	(112), (113)
unmodified and cationized spherical lignin nanoparticles (methods: softwood Kraft Lignoboost lignin or hardwood birch lignin solubilized in acetone and then added to deionized water, resulting in LNPs; water-soluble cationic lignin, prepared by reaction with glycidyltrimethylammonium chloride, was mixed with the LNP dispersion to yield cationic LNPs)	pharmaceuticals (acetaminophen, carbamazepine, diclofenac, ibuprofen, metformin, metoprolol, and tramadol)	electrostatic attractions, π–π interactions, hydrogen bonding, and hydrophobic interactions	maximum total adsorption of more than 25 mg/g by anionic LNPs and 30 mg/g by cationic lignin; maximum removal of 60% for metoprolol at concentrations below 10 mg/L, contact time of 60 min, pH 5 at room temperature	batch experiments: analyzed pH, 3, 5, and 8.5; analyzed temperature, room temperature; dose of nanolignin adsorbent, 1 mg/mL; contaminant concentration, 20 mg/L (mixture); surface area, not reported; total pore volume, not reported; regeneration methodology, not included in this study; reusability, not included in this study	(116)
cross-linked lignin or carboxymethyl lignin (methods: commercial lignin solubilized in NaOH, then cross-linked with epichlorohydrin, and reacted with chloroacetic acid)	pharmaceutical (ofloxacin)	monolayer chemical adsorption, hydrogen bonding, electrostatic attractions, and π–π and hydrophobic interactions	maximum adsorption capacity of 0.828 mmol/g (approximately 299 mg/g) in neutral solution (pH 7) at 25 °C	batch experiments: analyzed pH range, 2.0–11.5; analyzed temperature, 25 °C; dose of lignin adsorbent, 167 mg/L; contaminant concentration, 0.05–0.25 mmol/L; surface area, not reported; total pore volume, not reported; regeneration methodology, static experiments with NaOH solution and filtration, washing, and oven-drying steps; reusability, 5 adsorption–desorption cycles	(106)
chitin modified with Kraft lignin (methods: Kraft lignin activated in a H_2_O_2_ solution, in which α-chitin powder was soaked)	pharmaceuticals (ibuprofen and acetaminophen)	electrostatic interactions, π–π interactions, ion–dipole interactions, and hydrogen bonding	maximum removal of up to 94.2% with a drug concentration of 1000 μg/L, adsorbent dose of 0.2 g, and contact time of 120 min at 25 °C	batch experiments: analyzed pH range, 2–10; analyzed temperature, 25–35 °C; mass of lignin adsorbent, 0.05–0.5 mg; contaminant concentration. 250–2000 μg/L; surface area, not reported; total pore volume, not reported; regeneration methodology, static washing with various eluents (water, ethanol, and methanol) and filtration steps; reusability, 3 adsorption–desorption cycles	(115)
diverse types of LNP-based nanocomposites or hybrids (methods: several types of modified and unmodified lignin (e.g., commercial alkaline lignin, alkaline lignin from papermaking liquor, Klason lignin from palm kernel shell wastes, or cellulolytic enzyme lignin from corn stover) solubilized in solvents (e.g., ammonium hydroxide aqueous solution, polyethylene glycol, recyclable THF, or dimethyl sulfoxide) and diluted with deionized water (either by addition, acid precipitation or dialysis) to form LNPs; nanolignin combined with synthetic or natural polymers or with inorganic nanoparticles)	cationic (e.g., MB, safranin-O, basic red 2) and anionic dyes (acid scarlet GR)	electrostatic attractions, π–π interactions, hydrogen bonding, ion exchange, and van der Waals forces	maximum adsorption capacity of up to 176.49 mg/g by amine lignin coated Fe_3_O_4_ nanoparticles; 138.88 mg/g by hydrogel of LNPs grafted with poly(acrylic acid); 74.07 mg/g by chitosan/LNP composite; 806 mg/g by a MnO_2_-nanodots-modified lignin nanocomposite at 25 °C	batch experiments: analyzed pH range, 2–12; analyzed temperature, 25–50 °C; dose of lignin adsorbent, 0.1–1 g/L; contaminant concentration, 20–700 mg/L; surface area, up to 51.69 m^2^/g; total pore volume, up to 0.182 cm^3^/g; regeneration methodology, extraction with ethanol, distilled water, diluted HCl, or aqueous solutions at pH 2 or 12; reusability, 4 or 5 adsorption–desorption cycles	(105), (118), (120), (149)
magnetic lignin-based microspheres (methods: organosolv larch and poplar lignins esterified with maleic anhydride, dissolved in THF, and diluted with water or commercial alkaline lignin aminated with diethylenetriamine and subjected to a sol–gel procedure to yield microspheres; in both cases, Fe_3_O_4_ particles were embedded in the LPs)	cationic dyes (e.g., MB, RhB, and MG), anionic dye (e.g., MO), and heavy-metal ions (e.g., Pb^2+^, Hg^2+^, and Ni^2+^)	electrostatic interactions and van der Waals forces	maximum adsorption capacity of up to 155 mg/g for cationic dye (MG) at pH 8; 39 mg/g for anionic dye (MO) at pH 4; 55 mg/g for metal ion (Hg^2+^) at pH 5 and room temperature	batch experiments: analyzed pH range, 3–8; analyzed temperature, 25 °C; dose of lignin adsorbent, 0.5–14 g/L; contaminant concentration, 100–150 mg/L; surface area, not reported; total pore volume, not reported; regeneration methodology, extraction with distilled water and vibration or with an elution solution containing NaCl and NaOH (pH < 12); reusability, 3 adsorption–desorption cycles	(129), (130)
diverse types of pristine or functionalized bulk lignins and lignin-based composites (methods: freeze-dried nitrogen-containing wheat straw lignin, freeze-dried and low-temperature annealing (about 300 °C) aerogels of commercial alkaline lignin, hydrogels of LS graft polymerized with acrylic acid, cross-linked with *N*,*N*′-methylenebisacrylamide and initiated by laccase/*tert*-butyl hydroperoxide, poplar lignin treated with horseradish peroxidase, lignin/polyurethane foam prepared with lignin extracted from pine needles as polyol and reacted with diisocyanate, hardwood Kraft lignin/polyethylene highly porous composite prepared in a twin-screw extruder, hybrid composites of LignoBoost and CleanFlowBlack Kraft lignins with silica prepared by a sol–gel method in the presence of (3-aminopropyl)triethoxysilane and tetraethoxysilane, or commercial alkaline lignin dissolved in deep eutectic solvents and regenerated after precipitation with water)	cationic dyes (e.g., MB, RhB, and MG), anionic dyes (e.g., acid red 88 and MO), neutral dyes (e.g., mordant red 11), metal ion (Cu^2+^)	electrostatic interactions, hydrogen bonding, π–π interactions, pore filling, van der Waals interactions, ion exchange, and pore filling	up to 96% discoloration efficiency by wheat straw lignin and maximum adsorption capacity of up to 156.4 mg/g by physically cross-linked alkaline lignin aerogel; 2195 mg/g by LS/acrylic acid copolymer hydrogel; 210.2 mg/g by enzyme-modified lignin; 80 mg/g by lignin/polyurethane foam; 8.1 mg/g by Kraft lignin and 0.55 mg/g by Kraft lignin/polyethylene composite; 60 mg/g by lignin/silica hybrids; 551.05 mg/g by deep eutectic solvent-regenerated lignin	batch and continuous flow experiments: analyzed pH range, 2–10; analyzed temperature, 22–120 °C; dose of lignin adsorbent, 0.5–50 g/L; contaminant concentration, 50–2400 mg/L; surface area, up to 25.6 m^2^/g; total pore volume, up to 0.14 cm^3^/g; regeneration methodology, centrifugation and washing with water and weak acid medium, extraction with distilled water, HCl, NaOH, ethanol, methanol or dioxane; reusability, 4–20 adsorption–desorption cycles	(121), (122), (125), (127), (128), (150)–^152^
diverse LNP-based materials (methods: dried 21 nm spherical nanoparticles of alkaline lignin functionalized with polyethylenimine by Mannich reaction with formaldehyde and CS_2_, ca. 160 nm alkaline lignin-based nanotrap obtained by inverse-emulsion copolymerization with diethylenetriamine and formaldehyde in the presence of Span80, Kraft lignin/GO composite nanospheres prepared by self-assembly in THF/water systems using dialysis, freeze-dried magnetic GO/commercial lignin composite nanoparticles prepared by Mannich reaction followed by self-assembly of GO onto lignin-containing Fe_3_O_4_ nanoparticles, 10–20-nm magnetic nanoparticles of carboxymethylated organosolv lignin conjugated by epichlorohydrin with amine-functionalized Fe_3_O_4_ core/mesoporous silica shell, mussel-inspired magnetic nanoparticles of alkaline lignin cross-linked with 1-ethyl-[3-(3-dimethylamino)propyl)]carbodiimide and *N*-hydroxysuccinimide and conjugated with DA hydrochloride and then self-assembled with Fe_3_O_4_ nanoparticles, nanohybrid of cerium oxide nanoparticle-loaded aminated commercial lignin prepared by precipitation, or nanoadsorbent of alkaline lignin grafted with polyethylenimine and loaded with nanoscale lanthanum hydroxide)	metal cations (e.g., Ag^+^, Ni^2+^, Cu^2+^, Cd^2+^, Zn^2+^, Pb^2+^, Hg^2+^, Cr^3+^, and Th^4+^), oxycations (e.g., containing U^6+^), oxyanions (e.g., containing Cr^6+^ and P^5+^)	electrostatic interactions, van der Waals forces, hydrogen bonding, complexation, and ion/ligand exchange	maximum adsorption capacity of up to 392 mg/g for UO_2_^2+^; 396 mg/g for Th^4+^; 1057.95 mg/g for Ag^+^; 282.51 mg/g for Pb^2+^; 209.70 mg/g for Cu^2+^; 368.78 mg/g for Cr_2_O_7_^2–^; 110.25 mg/g for Ni^2+^; 44.56 mg/g for Cr^3+^; 65.79 mg/g for PO_4_^3–^	batch experiments in both single-component and multicomponent systems: analyzed pH range, 1–14; analyzed temperature, 25–45 °C; dose of lignin adsorbent, 0.05–1.0 g/L; contaminant concentration, 2–500 mg/L; surface area, up to 91 m^2^/g; total pore volume, 0.442 cm^3^/g; reusability, solutions of Na_2_CO_3_, NaHCO_3_, and EDTA as desorbents, reuse in antibacterial or electromagnetic wave absorption applications, or extraction with NaOH or HCl followed by filtration; up to 5 adsorption–desorption cycles	(60), (137), (140), (144), (153)–^156^
diverse lignin microparticle composites (methods: purified alkaline lignin extracted from sugar cane bagasse anchored with polyethylenimine and glutaraldehyde cross-linker by inverse emulsion polymerization to yield bowl-shaped particles in the microscale; 1045-μm Kraft LPs prepared with sulfosuccinate sodium salt/1,2-dichloroethane/epichlorohydrin and coated with polyethylenimine/glutaraldehyde, or hybrid composites of CleanFlowBlack Kraft lignin-coated nanoporous silica microparticles prepared by a sol–gel method)	metal cations (e.g., Cd^2+^ and Co^2+^), oxyanion (e.g., containing Cr^6+^)	ion exchange, electrostatic attractions, pore filling (physical adsorption), complexation, and precipitation	maximum adsorption capacity of up to 53.37 mg/g for Cd^2+^; 657.9 mg/g for Cr_2_O_7_^2–^; 18 mg/g for Co^2+^	batch experiments: analyzed pH range, 1–10; analyzed temperature, 20–60 °C; dose of lignin adsorbent, 1–2.8 g/L; contaminant concentration, 10–1000 mg/L; surface area, up to 92 m^2^/g (BET) or 536 m^2^/g (SAX); total pore volume, not reported; regeneration methodology, extraction by HNO_3_ solution, followed by neutralization with NaOH and washing steps with deionized water or desorption with either NaOH solution, distilled water, EDTA, HNO_3_ or HCl, or H_2_SO_4_; reusability, up to 6 adsorption–desorption cycles	(146), (157), (158)
extracted and functionalized bulk lignin (methods: pristine Kraft lignin precipitated from black liquor with HCl or sulfur dioxide, then washed and dried; or pristine Klason lignin from *Pinus elliottii* sawdust, modified alkaline lignin from sawdust or alkaline glycerol modified lignins from *Ailanthus altissima*; enzymatic hydrolysis lignin functionalized with diethylenetriamine and esterified with CS_2_; commercial alkaline lignin dually modified after reactions with CS_2_ and chloroacetic acid; corn cob alkaline lignin after phenolation, ammonization and sulfuration; carboxymethyl lignin prepared under microwave assistance and cross-linked with formaldehyde; or Kraft lignin extracted from black liquor by CO_2_ treatment followed by purification with H_2_SO_4_; or commercial lignin doped with N and Cu by a two-step procedure comprising Mannich reaction with triethylenetetramine/formaldehyde and adsorption of Cu^2+^; or hardwood organosolv lignin and softwood enzymatic hydrolysis lignin cationized with glycidyltrimethylammonium chloride)	metal cations (e.g., Pb^2+^, Zn^2+^, Cu^2+^, Cd^2+^, Ni^2+^, Hg^2+^, Cr^3+^, and Co^2+^), oxyanions (e.g., containing Cr^6+^, As^5+^, and S^6+^)	ion exchange, pore filling, electrostatic interaction, complexation, hydrogen bonding, and van der Waals interactions	maximum adsorption capacity of up to 137.14 mg/g for Cd^2+^ at 25 °C; 87.05 mg/g for Cu^2+^; 302.3 for Pb^2+^; 0.172 mmol/g (11.2 mg/g) for Zn^2+^; 0.102 mmol/g (6 mg/g) for Ni^2+^; 67.8 mg/g for Cr^3+^ at pH 5; 39.5 mg/g for (Cr_2_O_7_)^2–^ at pH 2; 7.7 mg/g for Co^2+^; 180 mg/g for Hg^2+^; 33.33 mg/g for CrO_4_^–^ and Cr_2_O_7_^2–^; 253.5 mg/g for HAsO_4_^2–^; 54 mg/g for SO_4_^2–^	batch experiments in both single-component and multicomponent systems: analyzed pH range, 1–13; analyzed temperature, 10–55 °C; dose of lignin adsorbent, 0.2–1 g/L; contaminant concentration, 2–200 mg/L; surface area, up to 3.13 m^2^/g; total pore volume, up to 0.0164 cm^3^/g; regeneration methodology, centrifugation and extraction with HCl, or HNO_3_ followed by NaOH under stirring, or HNO_3_ and filtration, or contaminant release with NaOH followed by electrochemical reduction, or freezing followed by aggregation/sedimentation and separation by decanting the adsorbent from the supernatant after melting; reusability, up to 10 adsorption–desorption cycles	(53), (93), (102), (103), (131)–^133^, (135), (136), (138), (139), (141), (142), (159)
diverse types of bulk lignin incorporated in composites, polymers, and hybrid materials (methods: freeze-dried composite hydrogel based on sulfomethylated commercial lignin that underwent ultrasonic-assisted free-radical polymerization process with acrylic acid in the presence of *N*,*N*′-methylenebisacrylamide (MBA) and potassium persulfate (K_2_S_2_O_8_), a composite hydrogel of sodium LS and carboxymethylated Sa-son seed gum polymerizes with acrylic acid in the presence of MBA and K_2_S_2_O_8_, softwood Kraft lignin polymerized with styrene in the presence of the surfactant dioctyl sulfosuccinate sodium salt and the initiator α,α′-azoisobis(butyronitrile), composite of lignin anchored with polyethylenimine in the presence of formaldehyde (Mannich reaction) and CS_2_, ternary composite of conducting polypyrrole, sodium salts of lignosulfonic acid, and anthraquinone-2-sulfonic acid produced by one-step galvanostatic polymerization, commercial Kraft lignin reacted with H_2_O_2_ and combined with α-chitin, a hybrid material of lignin and sol–gel-derived MgO–SiO_2_, a composite based on Polyphepan (commercial hydrolytic lignin) and magnesium hydroxide prepared by a alkaline hydrothermal procedure, a composite based on residue lignin branched with polyethylenimine in the presence of sodium laurylsulfonate/SA/epichlorohydrin, lignin/polyaniline composites prepared by the polymerization of aniline onto Kraft lignin, or composite of diethylenetriamine-modified alkaline lignin integrated with zirconium hydroxide)	metal cations (e.g., Pb^2+^, Ni^2+^, Cu^2+^, Cd^2+^, Co^2+^, and Zn^2+^), oxyanions (e.g., containing Cr^6+^ and P^5+^)	ion exchange, pore filling, electrostatic interaction, ion–dipole interaction, cationic−π interaction, complexation/chelating interaction, cation exchange, precipitation, hydrophobic interactions, and precipitation	maximum adsorption capacity of up to 344.85 mg/g for Pb^2+^; 145.14 mg/g for Co^2+^; 172.41 mg/g for Cu^2+^; 166.67 mg/g for Ni^2+^; 277.78 mg/g for Cd^2+^; 82.41 mg/g for Zn^2+^; 898.2 mg/g for Cr_2_O_7_^2^; 167.7 mg/g PO_4_^3–^	batch or bed adsorption experiments: analyzed pH range, 1–11; analyzed temperature, 25–45 °C; dose of lignin adsorbent, 0.2–10 g/L; contaminant concentration, 25–1700 mg/L; surface area, up to 214 m^2^/g; total pore volume, up to 0.13 cm^3^/g; regeneration methodology, centrifugation and extraction with HCl, or HNO_3_ followed by NaOH under stirring, or HNO_3_ and filtration, or distilled water, or sulfuric acid solution, or combined treatment by sequential extraction with HCl and recovery of Mg(OH)_2_ with MgCl_2_ and NaOH solutions, or NaOH as eluent; reusability, up to 10 adsorption–desorption cycles	(134), (143), (148), (160)–^168^

**Figure 3:**
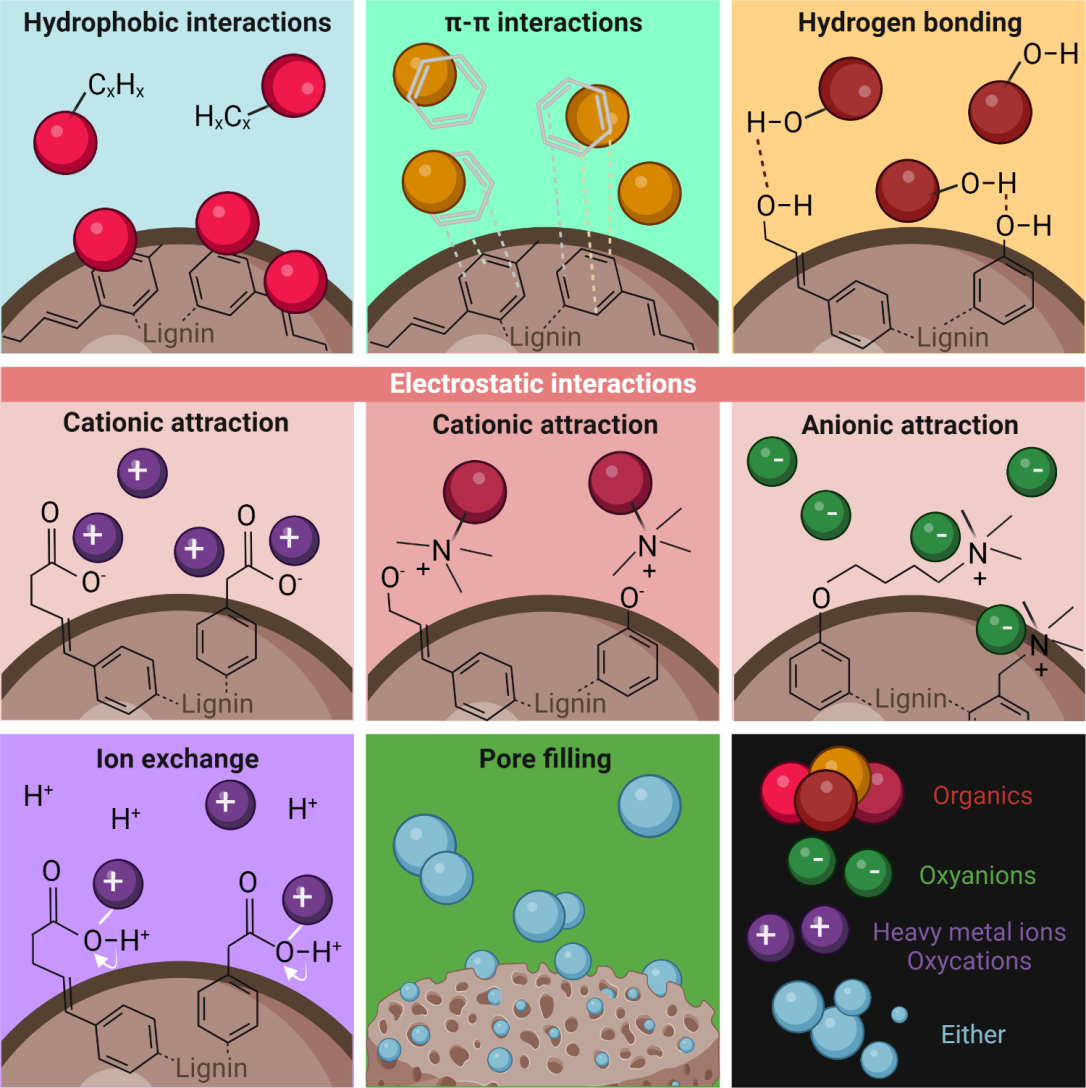
Depiction of the main mechanisms involved in the use of macro-, micro-, and nanostructured lignin-based adsorbents for the removal of contaminants from water. The mechanisms highlighted include hydrophobic interactions, π–π interactions, hydrogen bonding, electrostatic interactions, ion exchange, and pore filling. Due to hydrophobic interactions, pollutants containing nonpolar groups tend to aggregate in water and form nonspecific interactions with hydrophobic adsorbents to minimize their contact with water molecules. By π–π interactions, aromatic π-electron acceptors and donors attach in a stacking fashion. By hydrogen bonding, functional groups act as hydrogen donors/acceptors onto active sites of the adsorbents and contaminants. In electrostatic interactions, oppositely charged adsorbent surfaces attract cationic or anionic pollutants. In the ion-exchange mechanism, on the other hand, cations attached to the adsorbent surface are replaced by cationic contaminants, even yielding metal complexation. Finally, contaminants can also be absorbed by pore-filling mechanisms,^99^ when the high surface area and pore volume adsorbents promote the diffusion and trapping of molecules and atoms inside pores. Created in BioRender. Camargos, C. (2025) https://BioRender.com/o14b050.

As depicted in Figure 3, physical adsorption of pollutants by lignin-based adsorbents involves hydrophobic and π–π interactions, hydrogen bonding, electrostatic interactions (cationic or anionic attraction), ion exchange, and pore filling (or pore adsorption) events. Nonspecific hydrophobic interactions relate to the entropy-driven tendency of contaminants and adsorbents containing nonpolar groups to aggregate in water.^94^ Conversely, intermolecular π–π stacking occurs when contaminants and adsorbents share aromatic functional groups and are considered to be π-electron acceptors/donors. Hydrogen bonding is stronger than π–π interactions and forms between the adsorbent and adsorbates containing functional groups that act as hydrogen donors and acceptors. These three mechanisms can, in particular, explain the adsorption of organic contaminants by lignin adsorbents.

Electrostatic interactions proceed as Coulombic attraction between oppositely charged adsorbents and organic or inorganic adsorbates. The pH and ionic strength of the aqueous systems highly impact the adsorption efficiency.^95^ The ion-exchange mechanism encompasses the substitution of protons or positively charged ions on the surface of adsorbents by cationic contaminants, usually metal ions. Cation-exchange mechanisms will depend on the type of functional groups on the adsorbent surface^96^ and the size of the cationic target contaminants.^97^ Metal complexation can also take place through interactions with oxygen-rich functional groups and π-electron-rich domains^98^ on the lignin aromatic structure.

Pore filling, on the other hand, consists of a morphological phenomenon that is not a binding mechanism.^94^ A high surface area and excellent pore volume promote the diffusion of contaminants from the water toward the inner surface and into the pores of adsorbents.^99^ Although pore adsorption is primarily dependent on the morphological/topographical features of the porous adsorbents, the adsorption process can benefit from the presence of active surface sites and the establishment of attractive van der Waals forces.^100^

Chemical adsorption or chemisorption comprises irreversible, high-energy-demanding electron transfer between adsorbents and adsorbates.^101^ Previous studies have shown, for example, monolayer chemical adsorption mechanisms with the formation of coordinate covalent bonds between lignin adsorbents and complexed metal ions, such as Cd(II),^102^ Pb(II),^103^ and As(V),^53^ or organic dyes^104,105^ and pharmaceutical molecules.^106^ Conversely, physical adsorption or physisorption is driven by low-energy interactions, which favor desorption and reuse procedures. Despite the prevalence of physisorption, chemical and physical adsorption often occur simultaneously.^107^

Table 1 summarizes several target contaminants, multiple adsorption mechanisms, adsorption performances, conditions, and reusability parameters of different lignin adsorbents applied to water treatment. We reviewed the literature to address the application of macro-, micro-, and nanolignin materials, removing organic (chemicals, pharmaceuticals, and cationic and anionic dyes) and inorganic (heavy-metal ions and phosphate and sulfate ions) pollutants from water.

### 2.1 Lignin Adsorbents for the Removal of Organic Contaminants

Bulk and micro- and nanosized lignins have been extensively applied as adsorbent platforms for aromatic chemicals, multiple pharmaceutical molecules, and cationic and anionic dyes from wastewater. As shown in Table 1, the adsorption performance of chemically modified lignin materials involves multiple mechanisms.

Robust π–π interactions and pore adsorption of adsorbate molecules were reported by Sun et al.^59^ as the primary physisorption process behind the removal of bisphenol A (BPA) by a porous Friedel–Crafts alkylated corncob lignin. These endocrine-disrupting compounds were removed in quantities of up to ca. 400 mg/g.^59^ This high adsorption efficiency is justified by the large specific surface area of the lignin adsorbent (up to 1100 m^2^/g) and its hierarchical micro- and mesopore structures. In comparison, nonporous alkaline lignin/β-cyclodextrin microspheres cross-linked with epichlorohydrin showed even higher adsorption capacity for BPA (499.2 mg/g),^108^ although presenting a much lower specific surface area (1 m^2^/g) and a total pore volume of almost 75 times smaller than that of the Friedel–Crafts alkylated corncob lignin. The cross-linked lignin/β-cyclodextrin microspheres also exhibited a maximum adsorption capacity of 497.6 mg/g for 2,4-D.^108^ These reported adsorption capacities were considerably higher than the saturation adsorption amounts of typical adsorbents, such as biochar (216.6 mg/g),^109^ AC (93.59 mg/g),^110^ and graphene oxide (GO; 19.2 mg/g).^111^ These lignin/β-cyclodextrin microspheres produced by reverse suspension polymerization possess a multifunctionalized surface chemistry with O/N-containing functional groups, hydrophobic benzene rings, and internal cavities. In addition to π–π stacking and adsorption/wrapping into hydrophobic cavities (pore filling), the physicochemical features of the adsorbent enabled electrostatic interactions and ion exchange with 2,4-D molecules as well as hydrogen bonding, hydrophobic interactions, and van der Waals forces with BPA. These microspheres also showed remarkable reusability, with constant removal rates of up to 4 adsorption–desorption cycles, prompting regeneration steps by using anhydrous ethanol under ultrasonication.

Other studies report on using lignin-*graft*-acrylic acid copolymers^112^ and aminated lignin^113^ for the removal of aniline and 2,4,6-trinitrotoluene (TNT), respectively. Grafted commercial lignin-based copolymers displayed a maximum adsorption capacity of 79.1 mg/g for aniline, a value significantly higher than that of conventional AC (27.1 mg/g).^114^ Because the adsorption mechanisms for aniline and TNT were driven by electrostatic interactions and hydrogen bonding, modified lignin adsorbents showed regeneration for up to 8 repeated cycles. Adsorbent regeneration was conducted using eluents such as hydrochloric acid, ethanol, and acetone with recovery percentages as high as 95%. Regeneration and recovery yields of up to 80% were also observed for bulk lignin^106^ and lignin/chitin-based adsorbents^115^ when used to adsorb pharmaceutical contaminants.

In a different study, spherical LNPs and glycidyltrimethylammonium chloride-cationized LNPs were applied to adsorb several neutral (acetaminophen and carbamazepine), anionic (diclofenac and ibuprofen), and cationic (metformin, metoprolol, and tramadol) pharmaceuticals.^116^ The unmodified anionic and the cationized LNPs from softwood Kraft Lignoboost and hardwood birch lignins were able to interact with the seven aromatic and nonaromatic drugs in multianalyte systems through multiple mechanisms. The aromatic ring (π–π) stacking and electrostatic attractions likely contributed to the moderate adsorption of cationic, anionic, and neutral aromatic pharmaceutical molecules by the polyaromatic lignin-based nanomaterials. Moreover, LNPs moderately adsorbed neutral but highly aromatic carbamazepine (3.8 mg/g) through additional hydrogen bonding and hydrophobic interactions. Conversely, the adsorption of acetaminophen (0.3 mg/g), which was equally undissociated and neutral, was poor compared to that of carbamazepine. While the adsorption of acetaminophen was not hindered by competition with other highly aromatic and charged pharmaceuticals, its smaller size, inferior nonpolar character, and lower octanol–water partition coefficient likely contributed to its weak interaction with lignin nanomaterials. Even though the overall results evidenced a low adsorption capacity for LNPs, the authors^116^ suggested that anchoring these nanomaterials to a support material would enable sustainable and cost-effective adsorbents with the potential to remove a broad set of pharmaceuticals found in real wastewater effluents. Accordingly, the removal performance of LNPs was highly improved when they were immobilized in nanocellulose cryogels.^117^ For example, the adsorption capacity toward diclofenac increased from 11.8 mg/g in cationic hardwood and softwood LNPs^116^ to 350 mg/g in LNP-decorated cationic cellulose nanofibril (CNF) cryogel.^117^

A freeze-dried chitosan/LNP bionanocomposite showed an uptake efficiency of up to 85% for methylene blue (MB) at pH 7–9.^118^ The adsorption phenomenon was ascribed to hydrogen bonding, electrostatic interaction, and ion exchange between the cationic dye and deprotonated phenolic and aliphatic hydroxyl groups in lignin extracted from the palm kernel shell, as well as the amino and hydroxyl groups in the chitosan. In a similar approach, aminated Kraft lignin was cross-linked with sodium alginate (SA), forming hydrogels capable of removing up to 95% of MB (388.81 mg/g) at increasing pH values.^119^ The interactions likely resulted from hydrogen bonding and electrostatic adsorption of tertiary amine and positively charged sulfur (S^+^) of MB through deprotonated functional groups onto the lignin/SA hydrogel.

In another study, the removal of MB by spherical LNPs modified with MnO_2_ nanodots was found to be facilitated by electrostatic interactions.^120^ Corn stover lignin-rich residues, extracted through ammonia fiber expansion and enzymatic hydrolysis, were solubilized in dimethyl sulfoxide and underwent dialysis against pure water to produce LNPs. These LNPs reacted with KMnO_4_ under vigorous stirring to yield hybrid MnO_2_/LNP, which was then freeze-dried. The presence of the nanodots doubled the surface area from 24 in LNPs to ca. 52 m^2^/g in the nanocomposite. Therefore, the hierarchically structured materials presented an abundance of binding sites for MB adsorption by electrostatic interactions, resulting in an estimated maximum adsorption capacity as high as 806 mg/g for MnO_2_/LNP, which was greater than that reported for LNPs alone (391 mg/g) and unmodified bulk lignin (56 mg/g) under the same experimental conditions, considering the Langmuir adsorption model.

In general, the adsorption performance of bulk lignin-based adsorbents is moderate or inferior, compared to that of LNPs. For instance, Sipponen et al.^121^ extracted nitrogen-containing lignin from wheat straw by a sequential two-stage hydrothermal/aqueous ammonia pretreatment and used the freeze-dried product to adsorb MB with an efficiency of 84–86 mg/g. In contrast, superabsorbent hydrogels made of soluble LS^122^ or sulfomethylated lignin^123^ grafted with acrylic acid showed adsorption capacities against MB as high as 2195 and 777 mg/g, respectively. However, in these cases, the ratio of acrylic acid to LS was 8:1 to 14:1, indicating a significant contribution of carboxyl acid groups in the acrylic grafting as binding sites for the dye cations. In another study, hydrogels of poly(acrylic acid) cross-linked with laponite reached up to 3846 mg/g of adsorption capacity toward the same cationic dye.^124^

Porous pine lignin-based polyurethane foams could remove anionic methyl orange (MO) with an efficiency of 3.8 mg/g and cationic malachite green (MG) with an efficiency of 36.1 mg/g (maximum adsorption capacity: 80 mg/g in Langmuir adsorption isotherm) after a 120 min contact time.^125^ Also, polyurethane foam produced from dissolved enzymatic hydrolysis lignin reacted with diisocyanate-adsorbed MB and Rhodamine B (RhB) with efficiencies of 67.1 and 96.1 mg/g, respectively.^126^ In such systems, lignin played a leading role as a polyol source. Similarly, lignin/silica hybrid materials exhibited an adsorption capacity of ca. 42–60 mg/g toward MB.^127^ In binary anionic dye systems, magnetic calcium LS-based adsorbents adsorbed Congo red, Titan yellow, and Eriochrome blue black R with an equilibrium capacity of up to 160 mg/g.^104^ On the other hand, twin-screw extruded porous composites made of Kraft lignin and low-density polyethylene showed a much lower adsorption capacity (0.55 mg/g) toward MB when used in a fixed-bed column composed of a continuous-flow system.^128^

In comparison, the adsorption performance of nanostructured systems containing lignin magnetic nanoparticles was similar to that of magnetic LS-based hybrids and surpassed that of lignin magnetic microspheres. Sodium LS/Fe_3_O_4_ magnetic spheres, with an average diameter of 260 nm and featuring a rough, irregular surface morphology, exhibited a significant specific surface area (27 m^2^/g) and a maximum absorption capacity of 180 mg/g for MB.^54^ Magnetic lignin- and amine lignin-coated Fe_3_O_4_ nanoparticle adsorbents adsorbed both MB and acid scarlet GR with maximum efficiencies of up to 211 and 176 mg/g, respectively.^105^ The adsorption mechanism consists of π–π-stacking and electrostatic interactions. Similar mechanisms were involved in the adsorption by microspheres of diethylenetriamine-modified lignin combined with Fe_3_O_4_ hydrogel^129^ or esterified lignin embedded with Fe_3_O_4_ nanoparticles.^130^ Their maximum adsorption capacity toward cationic dyes, such as RhB, MB, and MG, varied from ca. 16 to 155 mg/g, while anionic dyes (e.g., MO) were removed at a maximum adsorption capacity of 39 mg/g. Either magnetic nanolignin- or microparticle-based adsorbents could be easily regenerated and reused due to the simultaneous response to pH changes and a magnetic field. These adsorbents exhibited high recovery/recycling ratios of >90% and sustained removal efficiencies of up to 98% after 3 cycles.

### 2.2 Lignin Adsorbents for the Removal of Inorganic Contaminants

Table 1 contains relevant information regarding the adsorption of inorganic contaminants from water. For the last two decades, lignin-based adsorbents have been extensively applied as potential candidates for the remediation of wastewater streams contaminated by multiple metal cations, oxycations, such as uranyl ion, or oxyanions, including sulfur, phosphorus, and chromium compounds.

The largest contingent of the literature deals with investigating adsorbent platforms based on pristine or modified bulk lignins extracted from different plants using different methods. For example, dried Kraft lignin extracted by acid precipitation from black liquor wastes of papermaking was applied as an efficient adsorbent for lead (Pb^2+^),^131^ copper (Cu^2+^), and cadmium (Cd^2+^) cations in multicomponent systems^93^ and multiple heavy-metal ions in single-component systems (e.g., Pb^2+^, Cu^2+^, Cd^2+^, Zn^2+^, and Ni^2+^).^132^ The mechanism of adsorption includes electrostatic attraction between the cation ion and deprotonated carboxylic and phenolic functional groups of lignin.^131,132^ It is essential also to consider that metal-ion precipitation in the form of hydroxides on the lignin surface can occur at alkaline pH conditions.^133^ In addition to intraparticle diffusion of the small cationic adsorbates within lignin pores, ion exchange can occur under optimum pH conditions, usually near pH 6. At this condition, protons and singly charged monatomic cations (e.g., H^+^ and Na^+^) are replaced by higher valence metal cations.^93^

Conversely, pristine and modified lignin was also investigated as a potential adsorbent for hexavalent chromium (Cr^6+^) present in negatively charged chromate (CrO_4_^2–^) and dichromate (Cr_2_O_7_^2–^) ions,^134^ pentavalent arsenic (As^5+^) in arsenate oxyanions (AsO_4_^3–^ or HAsO_4_^2–^),^53,135^ and phosphate (PO_4_^3–^) and sulfate (SO_4_^2–^) anions.^136^ In the case of these negatively charged ions, the adsorption efficiency is usually optimal at low pH due to the anionic attraction exerted by protonated carboxyl, phenolic, and aliphatic hydroxyl functional groups on the lignin adsorbent surface.^134^ Indeed, a higher concentration of protons implies a greater H^+^ association toward the oxygen-containing groups on lignin, and the adsorbent becomes positively charged at pH values below its point of zero charge.^137^ In contrast, at elevated values of pH, deprotonation of these functional groups takes place, and electrostatic interactions become repulsive.^56^ Moreover, partial dissolution of the lignin adsorbents can also occur at alkaline pH, decreasing their adsorption capacity.^53^ Although other mechanisms such as metal complexation^56^ and hydrogen bonding^135^ should be considered, the electrostatic interaction-driven adsorption of anionic species by lignin is primarily pH-dependent.

In addition to overall pH control, other strategies to enhance the adsorption performance of lignin-based adsorbents comprise its functionalization with macromolecules or the introduction of lignin in composite materials, in its bulk, microform, or nanoform. For instance, acid-precipitated bulk lignin from wood alkali glycerol delignification liquors presented a maximum adsorption capacity toward mercury cations (Hg^2+^) of 4.4–5.3 mg/g.^138^ Modified enzymatic hydrolysis lignin, containing sulfur and nitrogen functional groups, exhibited a much higher removal of 180 mg/g.^139^ The incorporation of soft C=S, C–S, and −NH groups, which have excellent electron-donating capability, favored the formation of chemical complexation of Hg^2+^ by strong metal–ligand bonds. Similar results were observed for spherical 200 nm LNPs functionalized with diethylenetriamine, cross-linked by formaldehyde, and esterified with carbon disulfide to incorporate soft thiol groups and borderline amine/imine groups on the surface.^60^ Due to the presence of dithiocarbamate functional groups (R_2_N–C–S–R′), the LNPs simultaneously captured multiple heavy-metal ions from water with an outstanding adsorption capacity of more than 99% toward all contaminants and a maximum adsorption capacity of 1058 mg/g toward silver ions (Ag^+^). The adsorbent acted as a coordinated nanotrap for both soft acids (e.g., Ag^+^, Hg^2+^, and Cd^2+^) and borderline acids (Pb^2+^, Cu^2+^, and Zn^2+^), with these cations being loaded at the atomic level according to the Langmuir model.^60^ The authors proposed the reuse of a silver-adsorbed LNP-based adsorbent as an antimicrobial agent to prevent solvent-consuming and pollution-producing regeneration processes. Du et al.^140^ also suggested the alternative reuse of self-assembled GO and lignin-containing iron oxide magnetic nanoparticles (MLNPs) as electromagnetic-wave absorption materials. The GO–MLNP hybrid material exhibited high adsorption capacities of 147.88 mg/g for Pb^2+^ and 110.25 mg/g for Ni^2+^, which were satisfactorily maintained at more than 85% for up to 5 adsorption–desorption cycles, ensuring high recyclability properties. Chemically modified bulk lignins showed notably higher adsorption toward Pb^2+^ ions than unmodified extracted lignins. The maximum adsorption capacity increased from 49.8 mg/g in pristine Kraft lignin^131^ and 89.5 mg/g (0.432 mmol/g) in lignin precipitated from black liquor^132^ to 130.2 mg/g in phenolated alkaline corn cob lignin functionalized with amino and sulfonic groups.^141^ A similar trend was observed for disulfide/chloroacetic acid dual-modified alkaline lignin^142^ and carboxymethyl lignin,^103^ which presented adsorption capacities against Pb^2+^ ions of 208.3 and 302.3 mg/g, respectively. Zhang et al.^142^ indicated that the surface area of the dual-modified lignin (3.3 m^2^/g) was higher than that of precursor alkaline lignin (1.67 m^2^/g), which is a beneficial factor for increasing the adsorption capacity for Pb^2+^.

Nevertheless, when discussing the potential recyclability of the modified lignin, the same authors reported a sharp decrease to roughly 30% in the removal efficiency of Pb^2+^ due to partial desorption of cations from active adsorption sites and alterations in the material structure. Cross-linked carboxymethyl lignin^56^ and phenolated lignin grafted with amino and sulfonic functional groups^66^ demonstrated outstanding removal efficiencies of more than 85% after 5 and 10 adsorption–desorption cycles, respectively.

The combination of lignin with polymeric and inorganic additives has also been shown to enhance the adsorption performance of heavy-metal ions to be removed from simulated wastewater. For instance, the incorporation of lignin at a relatively small concentration of 10% (w/w) into a poly(acrylic acid) hydrogel slightly improved its adsorption capacity toward cobalt ions (Co^2+^) from 95.1 mg/g in pure acrylic polymer to 98.3 mg/g in the lignin-containing hydrogel.^143^ Such behavior could be ascribed to carboxyl and sulfonic functional groups in lignin, which create a large number of active sites for Co^2+^ adsorption via electrostatic interactions and imply a more loose and porous structure in the hydrogel, which favors pore filling. In another study, triethylenetetramine-functionalized commercial lignin demonstrated boosted selectivity toward As^5+^ oxygen-containing anions in mixed solutions of oxyanions simulating polluted water through hydrogen-bonding interactions.^135^ This mechanism relies on the strong interaction between the −OH group on the HAsO_4_^2–^ oxyanion and −NH_2_ groups on the amine-functionalized lignin adsorbent, as elucidated by experimental results and the density functional theory simulation method.

The simultaneous presence of chloride (Cl^–^) and nitrate (NO_3_^–^) ions did not affect the removal of CrO_4_^2–^ ions by self-assembled Kraft lignin–GO composite nanospheres.^144^ Conversely, the presence of sulfite (SO_3_^2–^) and PO_4_^3–^ ions in the medium was detrimental to the selective removal of Cr^6+^ compounds due to a competitive effect influencing the electrostatic interactions between the chromate and protonated hydroxyl groups on the hybrid nanospheres under acidic (optimal) conditions. Nevertheless, in single-component aqueous systems at pH 2 and 45 °C, the maximum adsorption capacity of this composite toward Cr^6+^ compounds reached 369 mg/g. This adsorption performance is comparable to that of highly efficient cross-linked hydrogels of GO–polyethylenimine (443 mg/g) studied as a potential adsorbent for the removal of Cr^6+^ from contaminated wastewater.^145^ Because its backbone contains several reactive amine groups, polyethylenimine can readily attract oxyanions. In fact, Kraft lignin spherical particles coated with polyethylenimine showed a great Cr^6+^ maximum removal capacity of 657.9 mg/g, as estimated by the Langmuir monolayer adsorption model.^146^ Comparatively, experimental results showed that the adsorption rate increased from 120.1 mg/g in pure LPs to 186.3 mg/g in polyethylenimine-modified LPs. Such an improvement in the adsorption capacity is likely attributed to the combined contribution of lignin’s ability to interact with oxyanions and the introduction of a porous surface layer containing cationic amine groups. Therefore, at the active adsorption sites, Cr^6+^ ions could be adsorbed by electrostatic interactions and then partially reduced to Cr^3+^.^146^ Increasing the process temperature can also facilitate the reduction of chromium, enhancing the removal performance of lignin-based adsorbents.^147^

A pronounced contribution of the incorporation of lignin macromolecules into polymer matrixes has also been reported. Polystyrene, a hydrophobic polymer widely used to produce adsorbents for water treatment, presented a lower surface area (15 m^2^/g) compared to that of bulk Kraft lignin (24 m^2^/g).^148^ When 40% lignin was polymerized with styrene monomers via free-radical polymerization in an aqueous emulsion, the resulting adsorbent exhibited a surface area of 44 m^2^/g and a maximum adsorption capacity of 45 mg/g for the Cu^2+^ ions. In contrast, the removal efficiencies of pristine lignin and pure polystyrene were 22 and 15 mg/g, respectively. Through experiments in a quartz crystal microbalance with dissipation, the authors further investigated the mechanisms of Cu^2+^ adsorption in separate and combined systems. The smaller surface area of Kraft lignin and polystyrene alone restricted the effective physical adsorption, with diminished access to the pores by the cations. The removal of Cu^2+^ by lignin was attributed to ion–dipole interactions and coordination between Cu^2+^ and the phenolate, aliphatic hydroxyl, and carboxylate polar groups on lignin. Adsorption on polystyrene relied on cation−π interactions based on dispersive and hydrophobic interactions. The larger surface area of lignin–polystyrene materials potentially contributed to physical interaction and pore filling, which enhanced the adhesion of copper ions and synergically influenced the adsorption, especially by cation−π, due to the aromatic ring-rich structure.^148^

The growing literature on lignin-based adsorbents applied to wastewater remediation indicates that these materials interact with multiple contaminants through intricate and diverse physical and chemical adsorption mechanisms including metal-ion complexation. These biosorbents are usually able to be regenerated and recurrently recycled, as shown in Table 1. By employing functionalization or blending to achieve pH-responsiveness and modulable specific surface area, lignin-containing adsorbents can be tailored to facilitate the removal of highly selective contaminants coexisting in water. Furthermore, even unmodified lignin, from the nano- to macroscale, has demonstrated effectiveness in adsorption technologies, enhancing the appeal of utilizing cost-effective lignin-based adsorbents as straightforward strategies for environmental applications.

## 3 Photocatalytic Activity of Lignin-Based Materials for Water Treatment and Remediation

Advanced oxidation processes (AOPs) are revolutionary environmentally friendly technologies that can achieve multiple water treatment goals. These processes involve the formation of highly reactive species such as hydroxyl radicals (^•^OH), which can degrade organic pollutants very effectively. Among these AOP technologies, photocatalytic oxidation, which utilizes the redox ability of photocatalysts under specific light conditions, has been widely adopted.^169−171^ Photocatalytic oxidation uses light irradiation to excite photosensitive semiconductors to induce the formation of electron–hole pairs, whose charge carriers react with oxygen (O_2_), water (H_2_O), and hydroxyl groups to generate reactive oxygen species (ROS) such as hydroxyl radicals (^•^OH) and superoxide radical anions (^•^O_2_^–^). ROS are very oxidative and can degrade pollutants through hydroxyl addition, substitution, and electron transfer.^172−174^

Photocatalysis has emerged as a feasible technology for the degradation of various pollutants in fresh and wastewater.^175−177^ However, the rapid recombination of photogenerated electron–hole pairs significantly limits the energy conversion efficiency of photocatalysts.^173,174,176^ To address these bottlenecks for the practical application of photocatalysis, several modifications for boosting the performance of photocatalysts have been proposed.^178,179^ Chemical modification,^180^ hybridization between semiconductors,^181,182^ and coupling between semiconductors and carbonaceous materials^183^ have been reported as favorable strategies for improving the photocatalytic efficiency.^176,177^

The effectiveness of photocatalytic processes depends on the characteristics of the photocatalyst itself. Materials with high specific surface area and porosity tend to have a greater adsorption capacity, which can also lead to improved photocatalytic activity.^177^ Porosity can increase the surface area and create more pathways for the reactants to reach active sites in the photocatalyst. Meanwhile, the enhancement in electrical conductivity also increases the photocatalytic ability.^176^ A high electrical conductivity can facilitate charge transfer, which is essential for many photocatalytic reactions. Materials displaying these distinctive characteristics can capture and retain the reactants and intermediates involved in the photocatalytic reaction more effectively. The result is more efficient reactions and a higher yield of the desired products.^174,177^

Lignin is an abundant biomacromolecule containing many aromatic rings and phenolic hydroxyl groups that can absorb light in the ultraviolet–visible (UV–vis) spectrum.^57,184^ The carbon-rich and complex structure of lignin makes it a promising candidate for photocatalytic modification.^57^ Furthermore, its large surface area and unique ability to assemble with other molecules and chemical structures are essential for modification with other photocatalysts.^57^ Here, we discuss recent research efforts using lignin as a photocatalyst or as a constituent for developing sustainable and efficient photocatalytic systems.

There is still limited research on utilizing lignin as the primary component of photocatalysts to break down pollutants. As the understanding of the chemical composition of lignin continues to grow, a new generation of nonmetallic photocatalysts that harness the photocatalytic properties of lignin has emerged. Lignin extracted from renewable biomass can be converted into lignin-based photocatalysts with the potential to produce hydrogen peroxide (H_2_O_2_) and hydrogen, thereby contributing to the conversion of waste sources into valuable products.^57,184^

A previous study presents a novel approach for using lignin as a photocatalyst to form H_2_O_2_ by O_2_ reduction and H_2_O oxidation under visible light.^185^ Using two of the most readily available and commercialized lignin models, LS and Kraft lignin, the authors demonstrate that under visible light from a solar simulator (298.2 K, λ > 400 nm), photoactivated LS and Kraft lignin could gradually accumulate H_2_O_2_ at rates of 80 ± 20 and 160 ± 30 mM/g_cat._·h, respectively. Based on the analysis of various H_2_O_2_-producing lignin models, it was found that only lignin with a β-O-4 bond, which might be involved in O_2_ reduction, can complete the photochemical reaction.^185^ Overall, this study provides new insight into the potential of lignin as a photocatalyst for solar-powered catalysis. Using lignin as a photocatalyst not only offers a sustainable and environmentally friendly approach to producing ROS but also presents a new perspective for research in photocatalysis and the application of lignin in water treatment technologies.

**Figure 4:**
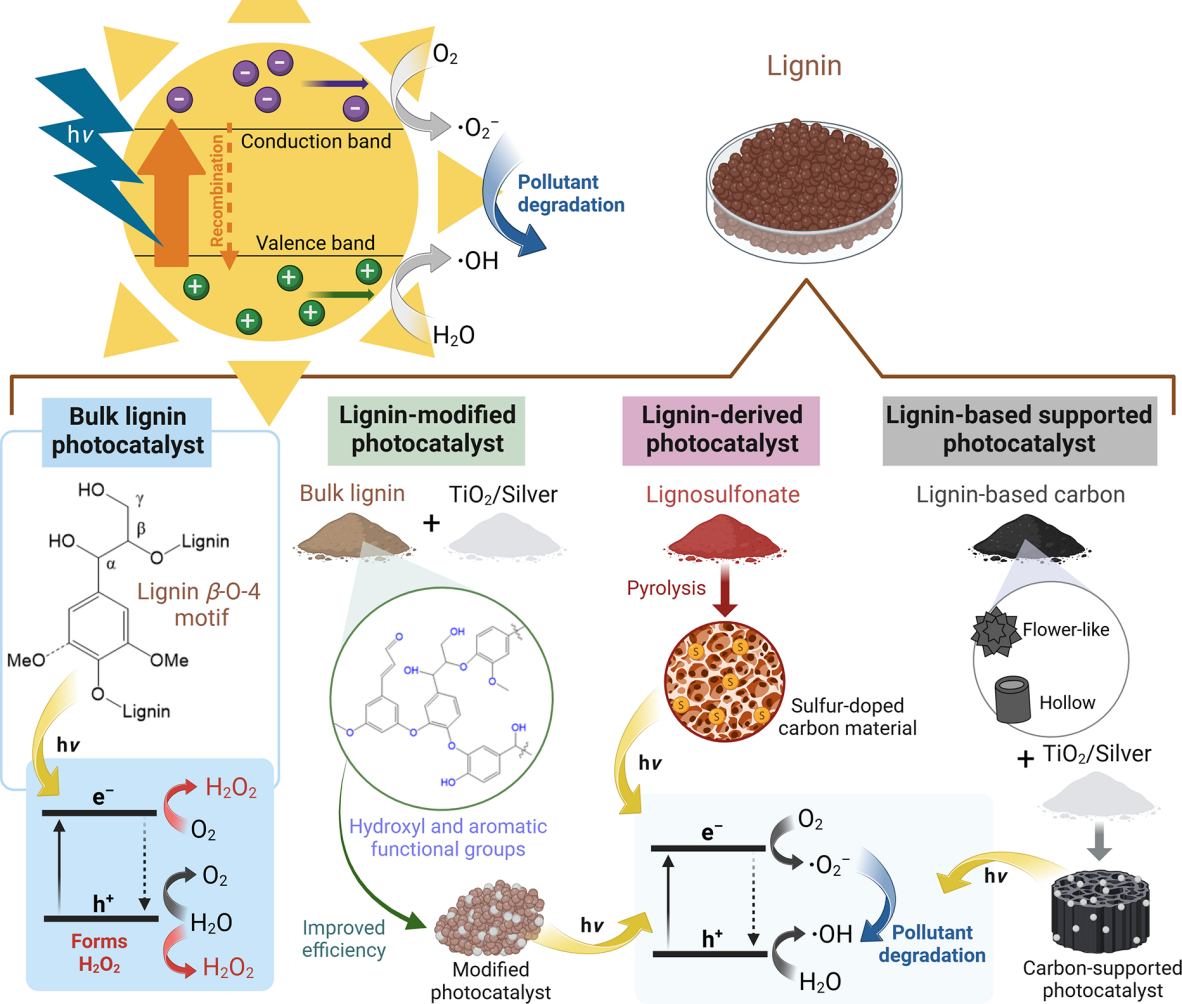
Scheme illustrating the different ways lignin is used to produce photocatalysts for pollutant degradation in water. β-O-4 bonds present in bulk lignin act as active sites for hydrogen production under light exposure. The combination of the benzene-rich structure of bulk lignin macromolecule with traditional photocatalysts may improve electron transfer and light absorption. LS lignin produced through the sulfite/sulfonation process is converted to sulfur-doped catalysts for pollutant degradation. Finally, LC materials with high surface area and abundant active sites are used as a supporting and conducive platform to anchor other photocatalysts. Lignin-carbon-supported photocatalysts present improved electron transmission efficiency and enhance the photocatalytic performance. Lignin demonstrates not only its versatility as a photocatalyst but also its potential to improve the efficiency and effectiveness of other photocatalytic materials. Created in BioRender. Camargos, C. (2025) https://BioRender.com/z26h235.

Figure 4 summarizes different approaches through which lignin-based materials have been investigated in photocatalysis applications. As outlined in Table 2, this aromatic macromolecule may be an excellent choice as either lignin-modified, lignin-derived, or carbon-supported photocatalysts aimed at the conversion and/or decomposition of biological contaminants, pharmaceuticals, chemicals, and dyes from water.

**Table 2:** Types of Lignin-Based, Lignan-Derived, or Carbon-Supported Photocatalysts, Target Contaminants and/or Application, Photocatalytic Activity, and the Experimental Conditions in Which These Photocatalysts Have Been Tested

photocatalyst	role of lignin	target application	photocatalytic activity	experimental conditions	refs
lignin photocatalyst	LS and Kraft lignin as photocatalysts (β-O-4 linkage functions)	formation of H_2_O_2_ by O_2_ reduction and H_2_O oxidation	photoactivated LS and Kraft lignin were able to gradually accumulate H_2_O_2_ rate production at rates of 80 ± 20 and 160 ± 30 mM/g_cat._·h	solar irradiation (λ > 400 nm)	(185)
modified photocatalysts	modification of TiO_2_ with hydroxyl and aromatic functional groups to enhance photocatalytic activity	selective oxidation of chemicals (benzyl alcohol to benzaldehyde)	when the benzyl alcohol conversion rate is 55%, the lignin-modified photocatalyst can selectively oxidize 97% of benzyl alcohol (BnOH) under UV light; in addition, the lignin-modified photocatalyst also exhibited excellent selectivity (100%) for moderate BnOH conversion (19%) under visible light	4 h under UV (375 nm) and visible (515 nm) light; 20 mL of 0.5 mM (0.01 mmol) benzyl alcohol solution; 20 mg of photocatalyst (1 g/L)	(190)
	induced dye binding to Ag by using LS aromatic functional groups with surfactant properties	degradation of dye (RY4G)	LS–Ag hybrid material exhibited significant photocatalytic activity in RY4G degradation, with a degradation efficiency of 87.6% after 3 h of irradiation	3 h of 365 nm UV-light irradiation; 5 mg of LS–Ag hybrid material; 100 mL of RY4G dye solution (20 mg/L)	(191)
	lignin as a support material introducing cyclic π bonds to facilitate charge transfer and increase the production of H_2_O_2_ through oxygen reduction and cooperate with carbon nitride tubes to release hydroxyl species	photocatalytic degradation of dye (brilliant black BN)	lignin-modified g-C_3_N_4_ nanotubes show the efficient removal of brilliant black BN (99.3%) after 60 min of illumination, with a degradation rate of 1.87 times that of the original carbon nitride tube	60 min of simulated daylight lamp (λ > 420 nm); 20 mg/L brilliant black BN 1 g/L photocatalyst	(192)
	acetylated lignin nanoparticles serving as catalyst carriers to improve the water dispersibility, stability, and reusability of porphyrin photocatalysts	photocatalytic degradation of pharmaceuticals (TMP and SMX)	porphyrin–lignin photocatalyst keeps nearly complete photodegradation of TMP and SMX after 24 h of irradiation with 7 cycles and has unprecedented TOC removal, namely, 75% and 85%	24 h of 400 W mercury lamp; SMX (2.16 mg, 8.5 × 10^–6^ mol) and TMP (0.45 mg, 1.5 × 10^–6^ mol); 2 × 10^–8^ mol of porphyrin–lignin photocatalyst	(187)
	lignin used as a supporting material, lignin also participating in the photocatalytic reaction process as an electron-transfer donor and acceptor, enhancing the photocatalytic activity	photodynamic antibacterial applications (*E. coli* and *S. aureus*)	porphyrin-conjugated lignin showed high photodynamic antibacterial activity, eradicating both Gram-negative and Gram-positive bacteria under 10 min blue LED light irradiation	10 min of blue LED light (450–460 nm)	(186)
carbon photocatalysts	production of sulfur-doped photocatalysts from LS as a carbon source	degradation of pharmaceutical (tetracycline)	sulfur-doped carbon photocatalyst prepared at 900 °C showed the highest photocatalytic degradation efficiency of about 70% after 3 h	300 W xenon lamp with 420 nm cutoff filter; 25 °C; 50 mg of the catalyst; 100 mL of a tetracycline solution (20 mg/L); initial pH of 7; after reaching dark adsorption equilibrium at 60 min, samples taken every 30 min	(195)
	sulfur-doped carbon photocatalysts prepared using sodium LS as the starting material	degradation of pharmaceutical (tetracycline)	carbon photocatalyst obtained at 300 °C has the highest efficiency (∼85%) for tetracycline after 120 min of light, which is nearly 40% higher than that of the carbon photocatalyst derived from alkali lignin	300 W xenon lamp with λ > 420 nm cutoff filter; room temperature, 100 mL of a tetracycline solution (20 mg/L), initial pH of 7; after dark adsorption equilibrium at 180 min was reached, reaction conducted under visible-light irradiation for 300 min	(196)
carbon-supported photocatalysts	lignin-derived CFs as carriers to load photocatalysts TiO_2_ and g-C_3_N_4_	degradation of dye (RhB)	under UV–vis irradiation, the degradation rate of RhB by the lignin/TiO_2_/g-C_3_N_4_ was able to reach 92.76%	180 min of illumination of 300 W UV–vis xenon lamp; RhB solution (30 mg/L)	(203)
	lignin-based CFs as support for TiO_2_ photocatalysts (CFs/TiO_2_) while providing the adsorption driving force and reducing the recombination ability of photogenerated electrons and holes	degradation of dye (MB)	CFs/TiO_2_ nearly degrade 100% MB after 24 min of irradiation, demonstrating a consistently high MB dye degradation activity of 91.5% even after 4 cycles of use	50 W xenon lamp (λ from 320 to 780 nm); 25 °C; 50 mg of the catalyst dispersed in 100 mL of a 50 mL MB solution with a stirring rate of 200 rpm or static state; samples taken every 3 min	(204)
	using lignin as a carbon support to produce LCZ catalyst with an excellent porous structure and specific surface area	degradation of dyes (MO and RhB)	LCZ photocatalyst, under simulated sunlight after 2 h of exposure, was able to degrade 92% of MO and 91% of RhB; the degradation activity was maintained at more than 80% after 5 cycles	500 W xenon lamp; 20 °C; 50 mg of the catalyst dispersed in 100 mL of a 50 mg/L MO or RhB solution; after dark adsorption equilibrium at 30 min was reached under visible-light irradiation	(205)
	LC nanosheets support the photocatalyst ZnO, providing a large specific surface area, avoiding ZnO aggregation, and improving light absorption	degradation of dye (MO)		simulated sunlight (300 W xenon lamp)	(206)
	modification of ZnO by XCL resulting in a larger specific surface area, improved charge transfer, and increased photocatalytic efficiency	degradation of chemical (4-CP)	under simulated sunlight, XCL/ZnO photocatalyst was able to degrade 85% of 4CP after 5 h, a 25% increase compared to pure ZnO	300 W artificial solar light lamp; 25 °C; 0.1 g of the catalyst were dispersed in 100 mL of a 10 mg/L 4CP; the photocatalytic process was carried out for 300 min	(207)
	LFC decorated with ZnO nanoparticles, which is beneficial for fast species transfer and high light-harvesting efficiency	degradation of pharmaceutical (sulfamethazine) and photocatalytic hydrogen evolution	ZnO/LFC photocatalyst exhibited excellent photocatalytic activities for the antibiotic sulfamethazine degradation and hydrogen evolution, which were 3.0 and 2.1 times that of pure ZnO, respectively; in addition, the recyclability of the composite photocatalyst is also significantly better than that of pure ZnO	500 W xenon lamp; room temperature; 20 mg of the catalyst dispersed in 50 mL of a 20 mg/L SMT; reaction conducted under visible-light irradiation; 50 mg of photocatalyst added to a mixed solution of water (180 mL) and triethanolamine (20 mL) and then transferred to a Pyrex reaction cell; photocatalytic hydrogen evolution performed with a 300 W xenon lamp	(209)
	LHC with abundant oxygen-containing functional groups and open structure allowing ZnO nanoparticles to be uniformly immobilized on the inner and outer surfaces of LHC, improving the photocatalytic efficiency	degradation of pharmaceutical (ciprofloxacin) and photocatalytic hydrogen evolution	ZnO/LHC showed higher photocatalytic degradation rate of ciprofloxacin and photocatalytic hydrogen production rate, which were 2.0 and 1.8 times that of pure ZnO, respectively	300 W xenon lamp (320 nm ≤ λ ≤ 780 nm); room temperature; 20 mg of the catalyst dispersed in 50 mL of a 20 mg/L ciprofloxacin; after dark adsorption equilibrium at 30 min was reached, reaction proceeded under visible-light irradiation; for photocatalytic hydrogen production, 50 mg of photocatalyst added to a mixed solution of water (180 mL) and triethanolamine (20 mL) and then transferred to a Pyrex reaction cell	(210)
	using lignin-based biomass carbon as a carbon source, CdS/LC photocatalyst prepared in combination with CdS nanoparticles to solve the problem of high agglomeration, showing excellent photocatalytic activity and high stability	degradation of pharmaceutical (ciprofloxacin) and photocatalytic hydrogen production	CdS/LC photocatalyst exhibits higher photocatalytic degradation rate and photocatalytic hydrogen evolution rate, which are about 2.3 and 3.73 times higher than that of pure CdS, respectively	500 W xenon lamp with UV cutoff filter (λ > 420 nm); room temperature; 30 mg of the catalyst dispersed in 100 mL of a 50 mg/L ciprofloxacin; after dark adsorption equilibrium at 30 min was reached, reaction proceeded under visible-light irradiation; for photocatalytic hydrogen production, 30 mg of photocatalyst taken and dissolved in 100 mL of solution (90 mL of water and 10 mL of lactic acid as the sacrificial agent)	(211)
	incorporation of LNRs into g-C_3_N_4_ nanomaterials to develop an efficient photocatalyst	degradation of chemical (triclosan)	in just 60 min under UV light (365 nm), LNRs/g-C_3_N_4_ photocatalyst degraded 99.90% of triclosan, and the hybrid catalyst maintained excellent cycling performance over 5 cycles	125 W xenon lamp (λ = 365 nm); room temperature; 25 mg of the catalyst dispersed in 50 mL of a 10 ppm triclosan solution; reaction under visible-light irradiation	(212)
	lignin used as a carbon precursor and support material for BiVO_4_; combined with lignin, inhibited charge recombination and accelerated charge transport, yielding high-efficiency photocatalysts	degradation of pharmaceutical (oxytetracycline hydrochloride)	degradation constant of oxytetracycline hydrochloride for the lignin-BiVO_4_ photocatalyst (011162 min^–1^) was 4 times that of BiVO_4_ alone (0.00285 min^–1^)	300 W xenon lamp (cut off λ < 400 nm); room temperature; 20 mg of the catalyst dispersed in 100 mL of a 10 mg/L oxytetracycline hydrochloride; after dark adsorption equilibrium at 60 min was reached, reaction conducted under visible-light irradiation	(213)
	novel BiOBr/lignin–biochar photocatalyst with oxygen-rich vacancies synthesized using organic capping molecules of lignin–biochar to enhance the activity of surface-ligand-induced catalysts	degradation of dye (RhB)	photocatalytic degradation efficiency of RhB by BiOBr/lignin–biochar was 99.20% at 60 min, much higher than that of pure BiOBr (44.50%)	300 W xenon lamp (with a 420 nm cutoff filter); 20 °C; 50 mg of the catalyst dispersed in 250 mL of a 30 mg/L RhB; after dark adsorption equilibrium at 60 min was reached, reaction conducted under visible-light irradiation	(217)
	lignin used as a precursor, providing a special synthetic environment for catalysts, resulting in a modified heterojunction Bi_4_O_5_Br_2_/BiOBr, which, in turn, prepares a highly efficient photocatalyst	degradation of dyes (MB and RhB)	composite photocatalyst shows excellent stability and could be reused for multiple cycles, achieving 85% degradation of RhB and 95% of MB after 100 min of illumination at 405 nm	20 W blue light (λ = 405 nm); 20 °C; 158 mg of the catalyst dispersed in 100 mL of 80 mg/L RhB or MB	(202)
	LC used as an adsorption material combined with a photocatalyst to solve the adsorption saturation problem and reduce electron pair binding, improving photocatalytic degradation capabilities	degradation of dye (MB)	composite catalyst was capable of degrading 99.9% of the 30 mg/L MB solution under 3 h of irradiation of UV and achieving 51.7% TOC removal	365 W UV mercury lamp (250 nm); 0.2 g of the composite catalyst; 30 mg/L MB solution	(218)

### 3.1 Lignin-Modified Photocatalyst for the Photodynamic Inhibition of Biological Contaminants

Li et al.^186^ produced porphyrin-grafted alkaline LNPs using THF as a solvent and water as an antisolvent. Employing lignin as a framework, porphyrin-conjugated lignin with significant photodynamic antibacterial activity was prepared by forming a p–n heterojunction through the coprecipitation method. Nanolignin constituted an encapsulation carrier to avoid aggregation and self-degradation of the photocatalysts. The p–n heterostructure of lignin and porphyrin reduces the catalyst band gap and promotes the reverse migration of charge carriers, thereby improving the photocatalytic performance. Not only that, the addition of lignin enhances the water compatibility of the catalyst and can efficiently generate ROS through the synergistic effect with porphyrin. Under the irradiation of blue LED light (450–460 nm), the composite catalyst was able to eradicate Gram-negative (*Escherichia coli*) and Gram-positive (*Staphylococcus aureus*) bacteria within 10 min.

### 3.2 Lignin-Modified Photocatalyst for the Degradation and Conversion of Organic Contaminants

To improve the stability and reusability of porphyrin photocatalyst to achieve sustainable photocatalytic applications, Piccirillo et al.^187^ used acetylated lignin nanoparticles as catalyst carriers to obtain a conjugated photocatalyst solid with good water dispersion. Using a 400 W mercury lamp as the irradiation source, this reusable stable photocatalyst promoted the nearly complete degradation of 0.1 mM trimethoprim (TMP) and sulfamethoxazole (SMX) after at least 7 cycles with an unprecedented total organic carbon (TOC) removal, namely, 75% and 85%, respectively.

Titanium dioxide (TiO_2_) is a semiconductor material that is primarily used in photocatalysis. TiO_2_ is known for its affordability and relatively low toxicity.^188,189^ Despite its advantages, TiO_2_ has some limitations that affect its applicability, such as its high recombination rate and low activity under visible light.^173,176^ In a recent study, Khan and collaborators^190^ reported that lignin, rich in hydroxyl and aromatic functional groups, can effectively improve the photocatalytic performance of TiO_2_. Modification of TiO_2_ with a chitosan-lignin composite led to enhanced photocatalytic activity under UV light. More specifically, after a 4 h exposure to UV (375 nm) light, the lignin-modified photocatalyst was able to selectively oxidize 97% of benzyl alcohol to benzaldehyde. At the same time, pristine TiO_2_ only achieved 82% within the same time frame. Further analysis utilizing electrochemical impedance spectroscopy revealed that the combination of chitosan–lignin and TiO_2_ led to a better charge separation efficiency and faster interfacial charge transfer in the lignin-modified TiO_2_ photocatalyst.

A recent study utilized sulfonate lignin, also known as lignosulfonate (LS), to modify nanosilver to achieve outstanding photocatalytic activity in the UV region.^191^ The LS–Ag hybrid material successfully decomposed reactive yellow 4G (RY4G) dye in the presence of H_2_O_2_ under UV irradiation at 365 nm (light intensity of 1000 μW/cm^2^). The aromatic structure of LS interacts with UV light, decomposing H_2_O_2_ and releasing hydroxyl (^•^OH) and hydroperoxyl (HO_2_^•^) radical species. The aromatic functional groups, which have surfactant properties, induced the binding of dyes to the photocatalyst, while silver acted as the photocatalyst itself. Combining the adsorption and catalytic properties was vital in achieving complete degradation of the RY4G dye. Their findings show that the Ag–lignin hybrid nanoparticles exhibited high photocatalytic activity and effectively degraded RY4G dye, with a degradation efficiency of 87.6% after 3 h of irradiation. The material also had good recyclability, showing significant photocatalytic activity in 2 cycles. This study demonstrates the potential of using lignin–silver materials as photocatalysts for wastewater treatment.

Graphitic carbon nitride (g-C_3_N_4_) holds potential in photocatalysis, but its widespread application is hampered by its low light absorption, small specific surface area, and slow charge dynamics.^192^ Zhu et al.^193^ prepared lignin nanospheres with large surface area through self-assembly to modify g-C_3_N_4_ nanotubes. The presence of lignin provided more photocatalytic reaction sites, and its abundant benzene ring structure conjugated with the triazine ring in the g-C_3_N_4_ nanotube to promote the adsorption and degradation of pollutants through π–π interactions. After modification with lignin microspheres, the specific surface area of the hybrid photocatalyst increased significantly from 290.5 to 374.5 m^2^/g, an increase of 28.9%. Photocatalytic experimental results show that under simulated daylight lamp (λ > 420 nm) irradiation, the addition of lignin can significantly improve the photocatalytic degradation efficiency of azo dyes. After 60 min of illumination, the removal efficiency of brilliant black BN is 99.3%, and the degradation rate is 1.87 times that of the original carbon nitride tube. The improvement of the photocatalytic performance is due to the cyclic π–π bonds in the lignin structure containing delocalized electrons. Induced by light, these delocalized electrons facilitate charge transfer and increase the production of H_2_O_2_ through oxygen reduction, accelerating the release of hydroxyl species by carbon nitride tubes.

The studies described using lignin as a physical/chemical modification material show that lignin helps the catalyst application and participates in the photocatalytic reactions as an electron-transfer donor and acceptor. This observation provides a powerful reference for the further development of lignin-modified catalysts with enhanced photocatalytic efficiency.

### 3.3 Lignin-Derived Carbon Photocatalysts for the Degradation of Target Contaminants

Nonmetal carbon materials have become a popular topic in photocatalysis due to their remarkable catalytic activity. The morphology of carbon nanomaterials plays a crucial role in determining their performance in photocatalyst modification.^174,177^ Heteroatom-doped graphene has emerged as a highly efficient, cost-effective, and environmentally friendly option for photocatalysis, making it a promising study area for researchers.^194^

Liu et al.^195^ utilized LS as a carbon source to produce sulfur-doped photocatalysts through pyrolysis to degrade tetracycline under visible light. The photocatalytic performance of the sulfur-doped carbon material was tested by measuring the tetracycline degradation efficiency under visible-light irradiation. The sulfur-doped lignocellulosic material prepared at 900 °C showed the highest degradation efficiency of ∼70% after 3 h exposure to a 300 W xenon lamp with a 420 nm cutoff filter. Further analysis revealed that the degradation of tetracycline was due to the generation of ROS, facilitated by the abundant presence of sulfur-containing chromophores such as sulfone and sulfonic acid groups. Their data indicated that the sulfonation process lowered the band gap of the porous carbon photocatalyst, preventing the recombination of photogenerated charge carriers and ultimately improving the photocatalytic activity of the sulfur-doped carbon material.

Another study conducted by Li and colleagues^196^ focused on the development of sulfur-doped carbon photocatalysts by carbonizing sodium LS at a low temperature. The photocatalytic performance of the photocatalysts was assessed by measuring their ability to degrade tetracycline under visible light. Their findings revealed that the carbon material obtained through carbonization at 300 °C exhibited the highest catalysis efficiency, degrading nearly 85% of tetracycline after 120 min of irradiation. This exceptional photocatalytic activity was attributed to sulfur doping, which increased the degradation rate of tetracycline by almost 40%. The research has provided valuable insights into the development and efficacy of sulfur-doped carbon photocatalysts derived from lignin. Utilization of lignin as a precursor to produce photocatalysts is a sustainable and cost-effective alternative to conventional photocatalysts.

### 3.4 Lignin-Derived Carbon-Supported Photocatalysts for the Degradation of Diverse Target Contaminants

Currently, most research on lignin photocatalysis involves using it as a carbon material to immobilize metal–semiconductor catalysts.^197−200^ Lignin is particularly well-suited for this purpose due to its high carbon content and customizable surface chemistry. These properties enable chemical modification and microstructure control, which are frequently employed to enhance the photocatalytic activity of the resulting lignin-containing materials.^201^ Furthermore, lignin-based carbon (LC) exhibits high specific surface area and strong electrical conductivity, resulting in efficient electron transfer and a significant increase in photocatalytic activity.^202^

Zhu et al.^203^ used lignin-derived carbon nanofibers (CFs) to support TiO_2_ and g-C_3_N_4_ for developing an efficient composite catalyst. Under UV–vis-light irradiation, the composite catalyst demonstrated 92.76% degradation of RhB, which was close to or superior to those of most reported carbon fiber-based photocatalysts. Another study showed that CFs produced from lignin have excellent hydrophobicity and high electrical conductivity.^204^ The integration of these lignin-based CFs with TiO_2_ resulted in improved light energy utilization and photocatalytic efficiency compared to pristine TiO_2_. When paired with the lignin-based CFs, TiO_2_ increased electron-transfer channels and charge separation while simultaneously improving its hydrophobicity. In fact, the lignin-based CFs/TiO_2_ demonstrated a contact angle of 130°, nearly triple that of pristine TiO_2_ (46°). This improvement in hydrophobicity increased the affinity of TiO_2_ for organic pollutants, allowing for a more efficient and faster degradation process. The experimental results showed that the degradation efficiencies of MB were 61.26% and 91.04%, respectively, for pristine CFs and TiO_2_ exposed to a xenon lamp for 30 min in a dynamic simulation experiment with a stirring rate of 200 rpm. On the contrary, CFs/TiO_2_ degraded 100% of MB after 24 min of irradiation. According to the pseudo-first-order kinetic model analysis, the rate constant of the CFs/TiO_2_ composite reaches 0.06973 min^–1^, which is 9.7 and 5.5 times those of CFs (0.00717 min^–1^) and pure TiO_2_ (0.01274 min^–1^), respectively. In comparison with pristine TiO_2_, CFs/TiO_2_ was 1.35 and 3.02 times more efficient in degrading MB under dynamic and static conditions, respectively.

CFs not only serve as supports for TiO_2_ photocatalysts but also provide a microporous and mesoporous structure for the adsorption and aggregation of MB. Due to the excellent conductivity of CFs, the photogenerated electrons on the conduction band of TiO_2_ are rapidly transferred to CFs, which significantly reduces the recombination reaction of photogenerated electrons and holes, further improving the photocatalytic efficiency and cycle life of CFs/TiO_2_ materials. In addition, the structure of the lignin-based CFs allowed for the semiembedding of TiO_2_ in the fibers, resulting in increased reusability and durability of the photocatalyst. The study surmises that the lignin-based CFs/TiO_2_ photocatalyst demonstrated a consistently high MB dye degradation activity of 91.5% even after using 4 cycles.

Zinc oxide (ZnO) has emerged as a popular photocatalytic material due to its stable performance and high quantum yield. However, to boost its effectiveness, researchers have developed a composite material called LC and ZnO (LCZ).^205^ This composite material was created by carbonizing a mixture of ZnC_2_O_4_ and quaternized alkaline lignin (QAL) in a furnace under N_2_.^205^ The LCZ composite has a porous carbonaceous structure that provides ample adsorption sites, resulting in a superior photocatalytic performance. By optimization of the LC and ZnO ratios (adding 4 g of QAL), carbonization time (2 h), and temperature (600 °C), LCZ displayed a maximum specific surface area of 317.765 m^2^/g, a pore volume of 0.4344 mL/g, and an average pore size of 1–30 nm, which offer an abundance of adsorption sites and greater light absorption while preventing ZnO agglomeration. Under simulated sunlight (500 W xenon lamp), the LCZ photocatalyst demonstrated exceptional photodegradation ability against anionic dyes. After 2 h of exposure to LCZ in simulated sunlight, 92% of MO and 91% of RhB present in an aqueous solution were degraded. The experimental results show that the LCZ photocatalyst has excellent cycle stability after 5 cycles, and the degradation rate can still be maintained above 80%. Tang et al.^206^ uniformly dispersed ZnO nanoparticles in the three-dimensional network structure of LC nanosheets. The modified catalyst increased the specific surface area while avoiding the aggregation of ZnO. At the same time, after high-temperature carbonization of lignin, the black background of lignin carbon and the C=C and C=O double bonds in the benzene ring structure made the composite catalyst exhibit better UV- and visible-light absorption. Under simulated 300 W sunlight, the hybrid catalyst was able to significantly degrade 96.9% of 15 mg/L MO within 30 min and maintain a stable performance within 5 cycles.

A LC xerogel/zinc oxide (XCL/ZnO) photocatalyst has been created by annealing and calcining a mixture of zinc nitrate, Kraft lignin, and microcrystalline cellulose.^207^ The resulting XCL/ZnO photocatalyst had a higher specific surface area (12.15 m^2^/g) than ZnO (5.17 m^2^/g) due to the combination with carbonaceous material in the form of nodular particles and smaller particle aggregates. The increased surface area of XCL/ZnO led to an improved photocatalytic efficiency, thereby reducing electron recombination. Under simulated solar light, the XCL/ZnO catalyst was able to degrade 85% of 4-chlorophenol (4CP) after 5 h, marking a 25% increase compared to pure ZnO. The addition of a Kraft lignin-based carbonaceous material also improved the overall reaction rate. XCL/ZnO demonstrated a reaction rate double that of pristine ZnO upon degradation of 4CP. Furthermore, XCL/ZnO 1.0 (0.75 g of Kraft lignin and 0.25 g of microcrystalline cellulose) displayed a constant photodegradation activity of 60%, greater than a counterpart catalyst made using tannin instead of lignin.^208^ This suggests that LC is more effective at strengthening the photocatalytic activity of ZnO than carbons produced from other carbon sources.

In another study, ZnO was modified with a lignin-derived flower-like carbon (LFC/ZnO), whose LFC portion was prepared from enzymatically hydrolyzed lignin (EHL).^209^ The unique flower-like structure of the lignin-derived carbon allows for even attachment of ZnO nanoparticles to the carbon nanosheets, preventing clumping and creating a large contact area for pollutants. The results of the study show that the LFC/ZnO photocatalyst degraded over 95% of the antibiotic sulfamethazine in 3 h of simulated sunlight exposure, while pure ZnO only degraded 63%. The photodegradation efficiency of the LFC/ZnO hybrid material was found to be 3 times higher than that of pure ZnO. Furthermore, the results of photocatalytic hydrogen evolution experiments are similar to those of the photocatalytic degradation experiments. The photocatalytic hydrogen production rate of the ZnO/LFC photocatalyst is 29.0 μmol/h, which is about 14.1 times that of pure ZnO (2.1 μmol/h). Hybridization with LFC effectively inhibited the photocorrosion and agglomeration of ZnO nanoparticles during the photocatalytic reaction. Therefore, the recyclability of ZnO was significantly improved, showing high stability after 5 consecutive cycles.

The same research group also investigated lignin-based hollow carbons (LHCs) modified with ZnO.^210^ The composites containing 5% (w/w) LHC exhibited the best retouching effect, allowing for a hollow structure with open pores where ZnO nanoparticles could evenly coat the inner and outer surfaces of the material, increasing the number of active sites available for photocatalytic reactions. The LHC/ZnO photocatalyst was observed to degrade 98% of the organic pollutant ciprofloxacin after 90 min of simulated sunlight exposure, compared to 81% degradation by pure ZnO. Furthermore, the photocatalytic hydrogen production rate of LHC/ZnO (52.2 μmol/h) was 1.8 times that of pure ZnO (28.5 μmol/h).

Cadmium sulfide (CdS) has been recognized as a highly efficient photocatalyst due to its exceptional visible-light absorption coefficient. Binding to LC has been performed to prevent agglomeration of CdS, thus improving its stability and photocatalytic activity.^211^ To assess the feasibility of this hybrid material, CdS nanoparticles modified with LC (CdS/LC) were prepared and tested for their ability to catalyze the degradation of ciprofloxacin and the production of hydrogen. The results indicated that the combination with LC significantly enhanced the photocatalytic efficiency and recyclability of CdS by considerably increasing the specific surface area from 1.8 to 111.5 m^2^/g. In only 60 min, the CdS/LC photocatalyst degraded 98% of the ciprofloxacin and exhibited a photocatalytic hydrogen evolution of 234.7 μmol in 4 h, around 2.3 and 3.73 times higher than that of pure CdS. Moreover, CdS/LC retained its stability even after 4 consecutive degradation cycles, indicating its durability for practical applications.

Analogously, g-C_3_N_4_ has become a popular choice for metal-free semiconductor photocatalysts due to its exceptional thermal stability. However, its fast charge recombination presents a challenge for its application. Researchers have addressed this issue by using lignin nanorods (LNRs) as electron-transfer channels in g-C_3_N_4_ to accelerate the rate of charge transfer.^212^ The resulting LNRs/g-C_3_N_4_ material demonstrated remarkable capabilities in degrading triclosan, a commonly used antimicrobial ingredient. Under UV light (365 nm) for just 1 h, LNRs/g-C_3_N_4_ degraded 99.90% of triclosan while maintaining excellent performance over 5 reuse cycles.

Bismuth vanadate (BiVO_4_), which responds well to visible light due to its narrow band gap (2.4 eV), has been combined with a porous material produced through the carbonization of lignin.^213^ The lignin–BiVO_4_ porous carbon material enhanced electron transfer and suppressed charge recombination in BiVO_4_. Additionally, the carbonized lignin was able to control the growth of the BiVO_4_ crystals and improve the surface-ligand-induced catalyst activity. The addition of lignin-based porous carbon improved the degradation ability of BiVO_4_ under visible light (300 W xenon lamp), particularly against broad-spectrum antibiotics. Specifically, the degradation rate constant for oxytetracycline hydrochloride was 4 times greater for lignin–BiVO_4_ (0.011162 min^–1^) than for BiVO_4_ alone (0.00285 min^–1^).

Bismuth oxybromide (BiOBr) is a photocatalyst with great potential for environmental applications, such as breaking down organic pollutants and producing hydrogen through water splitting.^214−216^ Yang et al.^217^ prepared BiOBr/lignin–biochar composites with varying ratios of BiOBr and lignin–biochar. They found that the addition of lignin–biochar significantly improved the photocatalytic activity of BiOBr. The optimal BiOBr/lignin–biochar ratio was found to be 2:1, resulting in a 99.2% degradation rate of RhB. Similarly, Budnyak et al.^202^ synthesized a hybrid material composed of hydrolyzed lignin and two semiconductors, Bi_4_O_5_Br_2_ and BiOBr. The lignin was extracted from wheat straw via acid-catalyzed hydrolysis and was used as a precursor. The hybrid lignin-containing material prepared via a facile solvothermal method exhibited excellent photocatalytic activity for the degradation of RhB and MB under UV–vis-light irradiation. Moreover, the hybrid photocatalyst shows excellent stability and can be reused for multiple cycles. The photocatalyst reduced the RhB concentration from 80 to 12.3 mg/L and the MB concentration from 80 to 4.4 mg/L after 100 min of illumination at 405 nm.

Similarly, adsorption materials and photocatalysts have been integrated to solve adsorption saturation and electron-pair binding problems during the photocatalytic process. For example, this integration has been applied to the ZnAl_2_O_4_/BiPO_4_ heterojunction catalysts. Li et al.^218^ took advantage of the large aromatic structure and high conductivity of LC to effectively improve the poor adsorption capacity of the ZnAl_2_O_4_/BiPO_4_ heterojunction catalyst, while inhibiting the recombination of charge carriers generated by light and enhancing the photocatalytic performance. Under irradiation of a 100 W UV mercury lamp (365 nm), 0.2 g of the composite catalyst can degrade 99.9% of the 30 mg/LMB solution in 3 h and achieve 51.7% TOC removal.

Utilizing lignin as a support material to develop hybrid photocatalysts has proven to be a promising route to improve the efficiency and stability of various photocatalytic materials. Through a series of innovative approaches, researchers have leveraged the unique properties of lignin to optimize the performance of semiconductor compounds. Integrating LC with materials such as TiO_2_, ZnO, CdS, g-C_3_N_4_, BiVO_4_, and BiOBr significantly enhanced the photocatalytic activity, electron-transfer efficiency, and charge separation.^180,183,200,206,209,210,213−216,219^ Various studies demonstrate the suitability of LCs as support materials, enabling the creation of composites that exhibit enhanced degradation capabilities for various pollutants. Furthermore, the sustained performance of these hybrid materials over multiple cycles highlights the durability and practical applicability of the developed photocatalysts. Overall, the synergistic combination of LCs with various photocatalytic materials holds great promise in addressing environmental challenges through efficient and sustainable pollutant degradation processes.

In the realm of photocatalytic technology, lignin-based materials offer a bridge between nature-based compounds and advanced oxidation processes. Combining the inherent properties of lignin, such as its aromatic structure, phenolic groups, and carbon-rich composition, with other semiconductors is a valuable strategy for solving issues related to environmental pollution. Through the various studies reported above, the incorporation of lignin into photocatalytic systems has shown promising results in addressing the challenges of rapid electron–hole recombination and low energy conversion efficiency. Whether as a carbon support material or a modification agent, lignin has demonstrated its capacity to enhance charge separation, increase surface area, and provide a conducive environment for efficient photocatalytic reactions. These innovations not only contribute to the effective degradation of diverse pollutants in water but also extend the application of lignin as a valuable resource in environmental remediation and sustainable materials development. Therefore, it is surmised that the utilization of lignin in photocatalysts can help advance water treatment technologies. Primarily, the use of lignin as a component for novel photocatalyst development not only provides a new method for designing and obtaining sustainable and environmentally friendly photocatalysts but also offers a novel angle for exploring and utilizing lignin as the central agent in photocatalytic processes. As researchers continue to refine and expand the applications of lignin-based photocatalysts, we anticipate further breakthroughs in pollutant degradation, waste utilization, and the pursuit of greener and more effective water treatment technologies.

## 4 Lignin-Based Flocculants for Water Treatment and Remediation

Lignin, in the form of solubilized bulk materials (e.g., oxidized, sulfomethylated, and polymerized) and dispersed nanoparticles, has been investigated as a sustainable and effective bioflocculant to remove biological, organic, and inorganic contaminants from water. The performance of these bioflocculants is comparable to that of traditional chemical flocculants, like ionic salts and water-soluble synthetic polymers.^12^ For example, in flocculation experiments, flocculants based either on commercial lignin or alkaline lignin from papermaking sludge removed around 90% of model dyes from simulated wastewater, producing large and resistant flocs.^220^ In a similar system, a conventional poly(aluminum chloride)/polyacrylamide (PAC/PAM) coagulant/flocculant achieved 95% color removal. Similarly, a poplar lignin-based flocculant synthesized by UV-light-initiated graft polymerization with (methacryloyloxyethyl)trimethylammonium chloride (DMC) exhibited a removal rate of 83.7–99.6% for *E. coli*, while the performance of some commercial flocculants, such as PAC/poly(diallyldimethylammonium chloride) (PDDA), was around 94%.^221^

Flocculation is a process that involves the agglomeration of colloidal particles, suspended solids, and dissolved inorganic and organic matter in water.^222^ This process occurs when particles and aggregates come into contact as a result of a destabilization process called coagulation.^223^ When particles are destabilized, they may collide with enough kinetic energy to overcome the barrier energy, which is the net interaction energy between attraction forces and repulsion arising from the overlapping of electrical double layers. Flocculants interact with particles, molecules, ions, and microorganisms by different forces (e.g., van der Waals, hydrogen bonding, and electrostatic attraction), resulting in agglomerates larger than the colloidal size that coalesce and precipitate and sediment in water. Conventional flocculation uses chemicals such as salts and polymers to facilitate the removal of contaminants by subsequent separation processes.

Separation processes face inherent challenges related to the chemical nature of the coagulant and flocculant and the cohesive forces between the flocs. Inorganic coagulants like PAC, lime (CaO), aluminum sulfate or chloride, ferric chloride, and ferrous sulfate^12^ generate large volumes of sludge, and their effectiveness highly depends on the water pH and temperature. Moreover, metallic residues of these cationic compounds in treated water are potentially harmful to human health.^224^ In conventional coagulation–flocculation, after the action of the inorganic coagulant, anionic or nonionic polymeric flocculants are used to agglomerate slow-settling, small flocs into larger, denser flocs. Similarly, synthetic polymer-based flocculants such as PAM, poly(ethyleneamine), and PDDA are used in direct flocculation protocols without coagulant aid. These petroleum-derived flocculants do not suffer from the limitations of inorganic coagulants, can be used in lower dosages, and yield large, compact, and strong flocs. However, they are expensive, not readily biodegradable, may release monomer residues, and potentially possess neurotoxic and carcinogenic properties.^12,224,225^ Considering the drawbacks of synthetic polymers, chitosan-, cellulose-, alginate-, and lignin-derived bioflocculants emerge as equally effective, more environmentally friendly, and cost-effective alternatives for partially or entirely replacing conventional flocculants in water and wastewater treatments.

**Figure 5:**
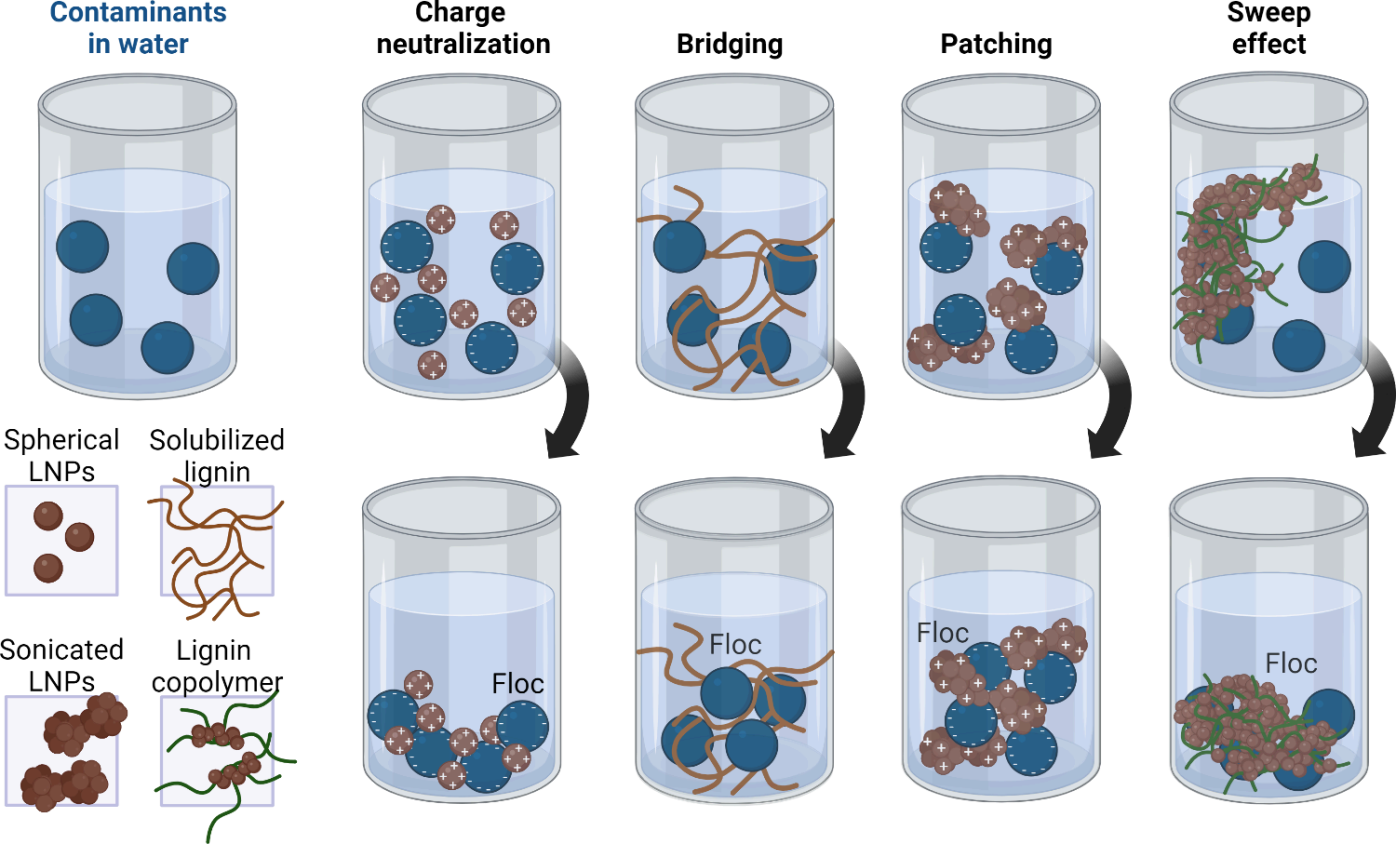
Depiction of different lignin-based materials used as flocculants and their typical application for removing charged and/or uncharged contaminants from water through four main mechanisms: charge neutralization, bridging, patching, and sweep effect. Charge neutralization occurs when charged lignin-based flocculants interact by electrostatic attraction with contaminant particles of opposite charge. In the bridging, modified lignin macromolecule chains connect with different contaminant particles, promoting their agglomeration. For patching, lignin flocculants attach to specific sites on the surfaces of the contaminant particles. These sites then interact with the sites containing opposite charges in adjacent particles. In the sweeping effect, polymerized lignin-based flocculants encase contaminant particles, and then larger structures drag uncaptured adjacent ones. Although electrostatic attraction is typically the primary mechanism in lignin-based flocculation processes, these three other mechanisms can also participate in the flocculation process. Note that the figure depicts only positively charged lignin and anionic contaminants in the electrostatic neutralization and patching mechanisms for simplification purposes. However, anionic lignin can remove cationic and nonionic contaminants from water as well. Created in BioRender. Camargos, C. (2025) https://BioRender.com/s09b610.

In water, particle agglomeration occurs through several mechanisms, namely, charge neutralization, bridging, sweep effect, and electrostatic patching, as illustrated in Figure 5. Charge neutralization is often the primary mechanism in the course when flocculants interact with the adsorption sites of oppositely charged contaminants. It involves the adsorption of counterions like mononuclear or polynuclear species, as well as polyelectrolytes, on the surface of the oppositely charged particles to be destabilized and flocculated.^223^ The interaction of oppositely charged species leads to a reduction of the resultant surface charges, measurable as a ζ potential near zero in the isoelectric point, and a decrease in electrostatic repulsion. This allows van der Waals attraction forces to promote the agglomeration of colloidal particles, molecules, ions, and fine suspended solids into flocs.^224^ In most real-world scenarios, negatively charged colloidal particles are prevalent, which makes positively charged flocculants like metal salts or cationic polyelectrolytes,^224^ including cationic-modified lignins,^226−228^ preferred for the charge neutralization approach.

Polymer bridging or interparticle bridging occurs when long polymer chains (high molecular weight) having low charge density adsorb onto the surface of two or more contaminant entities, either by chemical bonding or intermolecular interactions.^223,224^ The bridged particles then intertwine, generating larger flocs that settle better than those formed by charge neutralization.^229,230^ The efficiency of this mechanism depends on the length of the polymer chain and occurs when soluble modified lignin macromolecules are used to flocculate inorganics, such as colloidal particles^231−233^ and metal ions,^234^ and organic contaminants, especially cationic^235^ and anionic dyes.^226,236^

The electrostatic patch is formed when high-charge-density polyelectrolytes with low molecular weight partially bridge with contaminants that have a low density of charged sites. Therefore, oppositely charged patches in soluble polymerized^237^ and oxidized lignin,^238^ or in dispersed colloidal lignin systems,^239^ interact with charge domains in the free particles/entities through electrostatic attraction, which partially neutralizes the surface charges.^230^ The resulting flocs are weaker than those formed by bridging but stronger than those generated by charge neutralization only.^224^

The sweep effect occurs when suspended particulates are encapsulated by the flocculant, resulting in the formation of soft colloidal flocs.^240^ This process is also known as “sweeping” and can take place after the formation of bridging or patching networks, when clusters of bridged lignin networks capture small flocs and residual contaminants, particularly dyes^235^ and suspended solids.^241^

Table 3 provides a comprehensive overview of the primary targeted contaminants, flocculation mechanisms, and flocculation performance of different lignin systems applied to water treatment. The reviewed literature was organized and discussed to address the use of bulk and nanolignin for the removal of biological (including viruses and bacteria), organic (cationic and anionic dyes), and inorganic (clays and alumina) pollutants from water.

**Table 3:** Types of Lignin-Based Flocculants, Target Contaminants, Mechanism of Flocculation, Flocculation Efficiency, and Experimental Conditions under Which These Bioflocculants Have Been Tested

lignin-based flocculants	target contaminant	mechanism	flocculation efficiency	experimental conditions	refs
LNP–gelatin complexes (methods: LNPs prepared from switchgrass lignin by an ultrasonic-assisted alkali method; LNP dispersion directly mixed with a gelatin solution to produce LNPs–gelatin composites)	bacteria (*S. aureus* and *E. coli*)	charge neutralization, bridging, charge patching, and sweep effect	maximum flocculation efficiency of 98% at pH 4.5 using 2 g LNPs per 10^12^ cells	analyzed pH range, 2–6; analyzed temperature, N/A; dose of LNPs, 0–4 g/10^12^ cells; settling time, 0–40 min; recovery methodology, not mentioned; authors suggest that the large complexes flocculated with bacterial cells can be easily removed from water	(239)
spherical LNPs (methods: cationic spherical LNPs prepared by adding an acetone solution of softwood Kraft lignin into the water followed by surface modification with cationic bulk lignin)	viruses (cowpea chlorotic mottle viruses)	charge neutralization and hydrophobic effect	90–95% reduction in virus mobility and 80% virus removal using filtration or centrifugation	analyzed pH, 5; analyzed temperature, N/A; dose of LNPs, 0–200 mg/L; settling time, N/A; recovery methodology, LNPs form flocs with viruses that easily sediment or can be filtered/centrifuged and then burnt	(244)
water-soluble anionic lignin (methods: water-soluble fractions of softwood lignin oxidized with nitric acid or nitric acid/sulfomethylation)	cationic dyes (ethyl violet and basic blue)	charge neutralization (electrostatic), bridging, and sweep effects	up to 80% of dye removal at pH 7 using oxidized softwood thermomechanical pulping lignin; removal efficiency of 99% using sulfomethylated softwood Kraft lignin	analyzed pH range, 3–11; analyzed temperature, room temperature; dose of lignin, 100–300 mg/L; settling time, 2 h; recovery methodology, not mentioned; authors suggest that the large flocs are easily settled and can be removed by centrifugation	(235), (245)
different cationic lignin-based copolymers (methods: different bulk lignins (e.g., EHL from cornstalks, alkaline lignin or sodium LS from paper mill sludge) grafted with dimethylamine–acetone–formaldehyde, EDTA, dimethyldiallylammonium chloride and acrylamide, (2,3-epoxypropyl)trimethylammonium chloride, METAC, or a terpolymer of lignin and chitosan)	anionic dyes (e.g., acid black, reactive red, direct red, reactive turquoise blue, disperse red, disperse yellow, reactive blue, reactive orange, MO, and reactive black)	charge neutralization and bridging effect	99.5% dye removal	analyzed pH range, 4–9; analyzed temperature, room temperature; dose of lignin, 35–75 mg/L; settling time, 10 min –2 h; recovery methodology, not mentioned; authors suggest that separation could be performed by gravity filtration after settling	(55), (220), (246)–^248^, (254), (255)
cationic water-soluble lignin (methods: softwood Kraft lignin polymerized with METAC or with [2-(acryloyloxy)ethyl]trimethylammonium chloride or METAM)	clay suspension (kaolin particles)	charge neutralization, bridging, and patching effects	up to 96.4% turbidity removal	analyzed pH, 6 or 7; analyzed temperature, 25 °C; dose of lignin, 8–32 mg/g or 6–25 mg/L; settling time, 5–60 min; recovery methodology, not mentioned	(229), (250)–^252^
anionic water-soluble lignin (methods: Kraft lignin polymerized with 2-acrylamido-2-methylpropanesulfonic acid or oxidized Kraft lignin polymerized with acrylamide)	clay suspension (aluminum oxide particles)	charge neutralization (electrostatic interaction)	relative turbidity of aluminum oxide suspension was reduced from 1.0 to 0.2 or up to 86% alumina removal	analyzed pH, 7; analyzed temperature, N/A; dose of flocculant, 0–1.5 mg/g; settling time, N/A; recovery methodology, not mentioned	(61), (253)

### 4.1 Lignin Flocculants for the Removal of Biological Contaminants

Research on using lignin-based materials as bioflocculants to remove microorganisms from water is limited,^242^ as shown in Table 3. Nanocomposites based on ultrasonicated LNPs from switchgrass and gelatin have been investigated as flocculants for removing model bacteria (*S. aureus* and *E. coli*) from water.^239^

The LNPs–gelatin bioflocculant showed a remarkable flocculation efficiency of more than 95% at pH 4.5, which was faster than the flocculation behavior of aluminum sulfate and ferric chloride. As a comparison, aluminum sulfate and ferric chloride presented maximum flocculation efficiencies of 87% and 92%, respectively, for the removal of micro alga *Chlorococcum* sp.^243^ The LNPs–gelatin complex has a positive ζ potential below pH 5.0, making it possible to flocculate negatively charged bacteria by charge neutralization. However, the excessive addition of LNPs (more than 2.5 g of LNPs per 10^12^ cells) hindered this mechanism by increasing the negative charge in the complex, which promoted electrostatic repulsion between the flocculant and contaminants. Additionally, as the pH decreased to 4.0–4.5, increasing the concentration of LNPs in the system resulted in an increased flocculation efficiency, suggesting the contribution of charge patching, sweep, and bridging effects to the removal mechanism.

The removal of negatively charged viruses from water was achieved by using cationic and anionic colloidal softwood Kraft lignin with an average diameter of 100 nm.^244^ Charge neutralization and nonionic intermolecular forces were the main mechanisms driving the flocculation process. Anionic LNPs were produced using the nanoprecipitation approach, and quaternary amine-modified soluble lignin was adsorbed on the surface to yield positively charged nanoparticles. Because viruses are small in size, they are particularly challenging biological contaminants to remove from water. However, the LNPs showed a virus removal efficiency of up to 80%, indicating that lignin side streams can be used to produce cost-effective nanobased flocculants for large-scale water treatment applications. Besides being inexpensive and derived from abundant, biodegradable resources, the use of nanolignin-based flocculants allows for high removal rates of multiple biological contaminants while avoiding the production of secondary pollution.^242,244^

### 4.2 Lignin Flocculants for the Removal of Organic Contaminants

Bulk lignin has been widely applied as a bioflocculant for removing cationic and anionic organic dyes from wastewater, especially textile industrial effluents. Contrary to most inorganic flocculants, which are typically used in harmfully high concentrations, or even oil-based and synthetic commercial flocculants, which can be expensive and usually possess poor biodegradability,^230^ the utilization of lignin-based flocculants is a potentially cost-effective and environmentally friendly option for water and wastewater treatment.

Table 3 shows that most lignin-based flocculants used to remove dyes are derived from the functionalization of lignin extracted from lignin-enriched paper mill sludges. In studies conducted by Couch et al.^245^ and He et al.,^235^ soluble softwood bulk lignins were functionalized through nitric acid oxidation and/or sulfomethylation to enhance their anionic character. This resulted in an exceptional flocculation efficiency of up to 99% for cationic dyes, achieved through neutralization and bridging mechanisms. Although pH-dependent, the flocculation performance remained higher than 50% at a wide pH range (5 to 11), due to the process driven by the combination of charge neutralization, bridging, and sweep mechanisms. These findings demonstrate the potential of utilizing lignin-containing wastes for the development of value-added commercial products that are useful to water treatment processes.

Lignin has also been used to flocculate a broad set of anionic dyes under laboratory conditions, mimicking wastewater streams. Lignin isolated from the residue of enzymatically hydrolyzed cornstalks was grafted with dimethylamine to generate flocculants that remove azo dyes by 99% at low pH values and with a relatively long settling time.^246^ This modified lignin also has the benefit of reducing the amount of sludge produced by approximately 10% compared to conventional commercial flocculants such as PAC.^246^

Many other studies have reported the use of lignin extracted from papermaking or textile industry effluents.^55,226,228,247,248^ One approach involves the copolymerization of lignin-based flocculants with (2,3-epoxypropyl)trimethylammonium chloride.^247^ Other studies show that solutions of lignin grafted with dimethyldiallylammonium chloride/acrylamide^248^ and combined with PAC^55^ were able to remove color from water at an efficiency of around 90%, resulting in large, strong, and highly recoverable flocs. Softwood Kraft lignin grafted with [2-(methacryloyloxy)ethyl]trimethylammonium chloride (METAC) rendered high-molecular-weight and effective flocculants able to remove up to 60% of TOC from municipal wastewater.^227^ Similarly, a dual approach using sequential flocculation steps with anionic and cationic Kraft lignins polymerized respectively with acrylic acid and [2-(methacryloyloxy)ethyl]trimethylammonium methyl sulfate (METAM) proved highly efficient in removing up to 92.5% of the turbidity in peptone model wastewater.^249^ Such removal processes were sustained by charge neutralization and bridging mechanisms.^227,249^

### 4.3 Lignin Flocculants for the Removal of Inorganic Contaminants

Among inorganic contaminants, kaolin (clay mineral, Al_2_O_3_·2SiO_2_·2H_2_O) particles are commonly found in wastewater effluents from papermaking and mineral industries.^250^ Due to their small size and negatively charged surface, kaolin particles form stable and highly turbid colloidal suspensions. In previous studies, lignin has been polymerized with cationic monomers (e.g., METAC or DMC) to produce water-soluble cationic lignin-based copolymers that can interact with kaolin particles by charge neutralization, bridging, and patching mechanisms. The cationic lignin-based copolymers reduced turbidity by rates varying from 80 to more than 95%.^229,250−252^

Conversely, Kraft lignin has been polymerized with multigroup amide or acrylamide monomers to create water-soluble anionic copolymers based on lignin^253^ or oxidized lignin.^61^ These lignin-derived flocculants have been used to flocculate positively charged aluminum oxide particles, which are known to impart high turbidity to industrial wastewater. The lignin-based copolymers were able to eliminate up to 86% of the aluminum oxide^61^ and reduce turbidity by 80% using only 0.40 mg of flocculant per 1 g of alumina. A comparable concentration of the corresponding homopolymer of 2-acrylamido-2-methylpropanesulfonic acid was required to promote a similar flocculation performance.^253^ As a result, lignin can be used as flocculation matrixes or as partial components/substituents in consolidated commercial flocculants, improving their green attributes and broadening their functionality.

Thus, the utilization of lignin can help to reduce the quantity of synthetic monomers in conventional flocculants without compromising the efficiency of flocculation. Furthermore, branched lignin copolymers are particularly effective in removing color, promoting sedimentation, and improving the strength, size, and recoverability of flocs. Lignin-based flocculants are versatile and can be used over a wide range of pH levels and in lower doses than inorganic flocculants, resulting in less sludge production. However, because lignin can have varying molecular weights and surface chemistries, it requires thorough characterization and customization before use. Additionally, the potential use of lignin in more complex simulated wastewater systems^227,245^ needs further investigation to enable its commercial use for different types of contaminants.

## 5 Lignin-Based Materials for the Enhancement of Membrane Filtration Processes in Water Treatment and Remediation

Membrane filtration processes will be vital for maintaining current potable water supplies and expanding access to clean water through water reuse and desalination.^256−258^ This is because membrane-based water filtration processes can be used to separate a wide array of contaminants from water, including dyes, heavy metals, pharmaceuticals, and other contaminants of emerging concern.^259^ Furthermore, nanofiltration (NF) and RO filtration are effective for desalination and can be applied to address saltwater intrusion in aquifers and supplement water supplies with seawater.^256^ Membrane filtration processes are promising solutions to many of the challenges of clean water production; therefore, efforts should be made to overcome the practical limitations preventing the widespread implementation of these technologies.

One impediment facing membrane-based filtration processes is the propensity for membrane fouling caused by the inorganic salts, particulates/colloids, organic compounds, and bacteria present in the feedwater.^9^ Membrane fouling can, therefore, be separated into several types based on the causative contaminant: (1) scaling, (2) particulate/colloidal fouling, (3) organic fouling, and (4) biological fouling. Scaling is caused by the precipitation of inorganic salts on the membrane surface. Particulate and colloidal fouling occurs when particles that are too large to pass through the membrane accumulate on the membrane surface. Both scaling and colloidal fouling can be mitigated through appropriate pretreatment of the feedwater used for the membrane filtration process.^260^ Organic fouling caused by the adsorption of species such as proteins, polysaccharides, and natural organic matter, among other compounds, negatively impacts the performance of the membrane by reducing water permeability.^261^ Adsorbed organic matter can also act as a conditioning layer for bacteria, allowing them to more easily attach to the membrane surface, replicate, and develop into a biofilm.^262^ Organic fouling and biofouling are considered the most detrimental to membrane operation because they can be difficult to prevent, and the damage caused can be irreversible.^10^ For example, chlorine (readily available at most water treatment plants) cannot be used as a preventative measure for biofouling because some parts of the membranes are sensitive to oxidants and degrade in their presence.^263−265^ As a result, strategies to minimize biofilm formation on membranes have focused on either preventing the attachment of bacteria to the membrane surface or deactivating the bacteria upon contact with the membranes. Here, we highlight recent efforts focused on improving the membrane lifespan, antifouling properties, and efficiency of the membrane filtration process through the application of lignin.

### 5.1 Lignin-Based Modification of Semipermeable Polymeric Membranes

**Figure 6:**
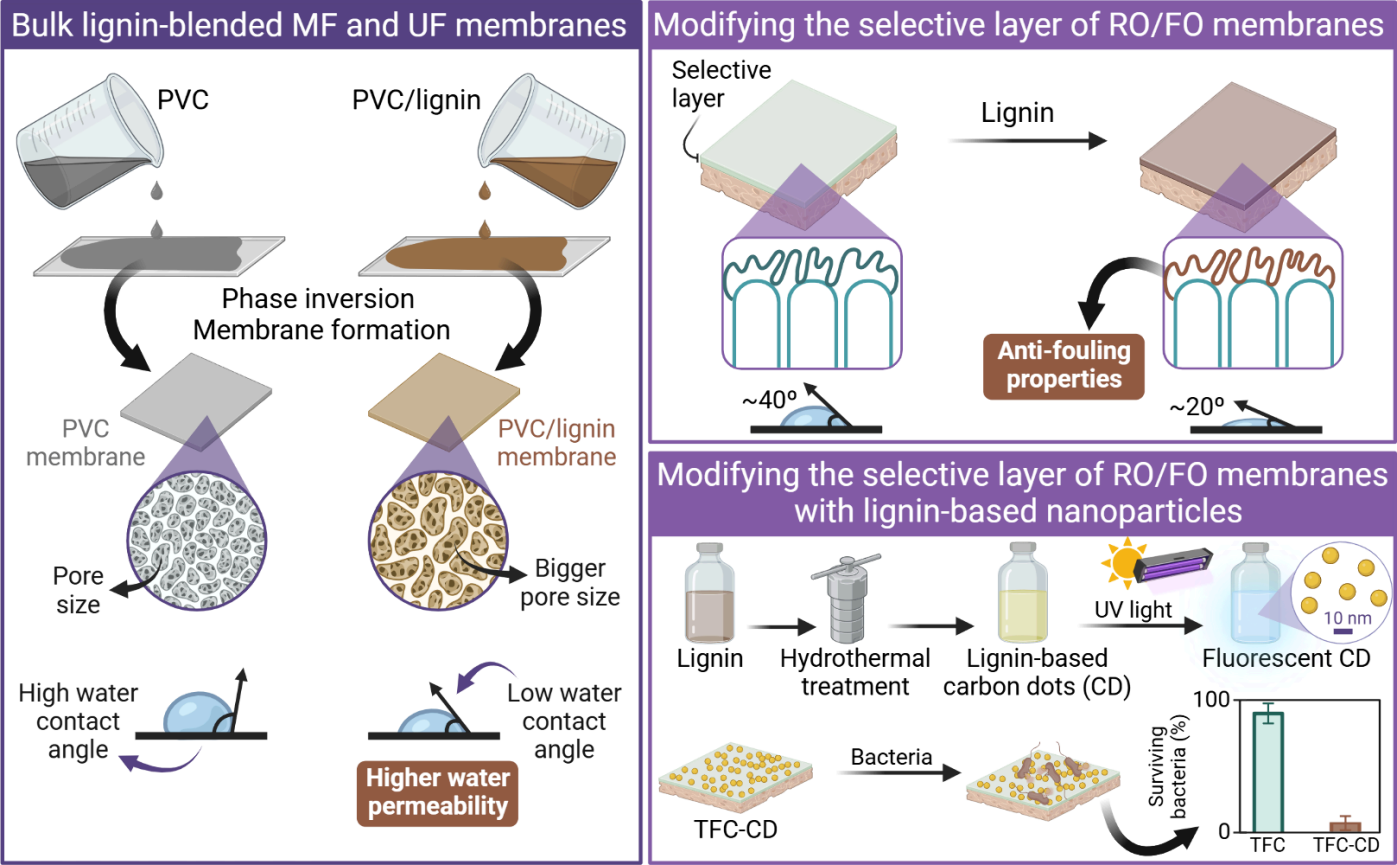
Scheme illustrating the different strategies used to modify filtration membranes with lignin-based materials. Blending bulk lignin with PVC forms MF and UF membranes with improved pore size and lower water contact angle compared to pristine PVC membranes; i.e., PVC/lignin membranes show higher water permeability than unmodified membranes (depiction inspired by the results reported by Yong et al.^272^). Modifying the selective layer with lignin enhanced the antifouling properties of TFC RO/FO membranes by increasing their hydrophilicity (depiction inspired by the results reported by Zhou et al.^273^). Additionally, lignin-based nanoparticles, such as fluorescent CDs, have been attached to the polyamide selective layer to impart antimicrobial and antibiofouling properties to RO/FO membranes under exposure to UV and simulated daylight (depiction inspired by the results reported by Yang et al.^17^). Created in BioRender. Camargos, C. (2025) https://BioRender.com/t76u396.

Semipermeable membranes composed of polymers such as poly(ether sulfone) (PES) and poly(vinyl chloride) (PVC) are commonly used for membrane filtration processes such as microfiltration (MF) and ultrafiltration (UF). MF and UF rely on a relatively low applied pressure to filter water through size-exclusion-based mechanical sieving and adsorption.^266−268^ While the structure of these polymeric membranes allows for both high water permeability and the retention of small contaminant molecules,^269^ further work is ongoing to overcome the permeability–selectivity trade-off for these semipermeable membranes.^270^ A common technique for improving the membrane performance is to produce a composite membrane by blending different materials into the membrane substrate. Lignin is favorable for this application because it can improve the membrane hydrophilicity,^271^ thereby improving membrane permeability (Figure 6).

Yong et al.^272^ produced lignin-doped PVC UF membranes with improved hydrophilicity and antifouling properties relative to pristine membranes using commercial lignin and a standard phase-inversion method. As the concentration of lignin blended in the PVC membrane increased, the water permeability of the membrane also increased when filtering both water–oil emulsions and humic acid (HA) solutions compared to the control PVC membrane. The highest increase in water permeability (347.26 L/m^2^·h) was observed for the Lig-5 membrane (containing a 1:2 ratio of lignin to PVC), which was nearly 3 times that of the control PVC membrane (111.60 L/m^2^·h). This could be partially attributed to the increase in hydrophilicity observed for the lignin-blended membranes, which had a water contact angle as low as 41.53° for the Lig-5 membrane, significantly lower than that of the control (106.73°). The increased porosity (84.9%) and mean pore size (25.5 nm) of the Lig-5 membrane relative to the control (76.0% porosity and 19.7 nm mean pore size) could also contribute to the improved permeability. In addition, higher retention of oil, suspended solids, and compounds contributing to chemical oxygen demand was observed for all membranes containing lignin compared with the control PVC membrane. An increase in hydrophilicity followed by an improved water permeability was also verified for poly(vinyl alcohol) (PVA) membranes prepared using water-soluble ozonated lignin as an additive^274^ and for membranes of polycaprolactone (PCL) blended with SKL in acetic acid.^275^

Biodegradable PCL/SKL electrospun membranes, specifically designed for the gravity-driven separation of oil/water emulsions, exhibited superhydrophilicity and underwater superoleophobicity, achieving separation efficiencies of 97–99%. Additionally, these membranes delivered impressive pure water flux rates of 800–900 L/m^2^·h and emulsion flux rates of 170–480 L/m^2^·h.^276^ The membrane containing 10% (w/w) SKL displayed excellent recyclability and reusability, sustaining an approximately 98% separation efficiency after 10 cycles. Moreover, the PCL/SKL membranes maintained adequate stability and consistent wettability across a wide pH range from 1 to 10. However, prolonged exposure to pH 12 resulted in hydrolysis, solubilization, and leaching of SKL, which adversely affected the structural integrity and functionality of the membrane.^276^ These results emphasize the potential of using lignin to enhance the filtration performance of MF and UF membranes, although β-O-4 bonds in lignin are prone to cleavage under highly alkaline conditions.^277^ In addition, the inherent heterogeneity of lignin can lead to variability in the performance and challenges during membrane fabrication. Furthermore, while lignin-doped membranes, such as AL/polysulfone (PS), demonstrate significant thermal stability,^278^ this stability may be diminished as lignin decomposes slowly over a broad temperature range (200–500 °C).^279^ This limitation may prevent the application of these membranes to the treatment of high-temperature oily wastewaters.

Mechanical strength and durability are also important factors to consider when designing membranes because UF and MF require an applied pressure during the process. In addition to improving the antifouling capabilities and hydrophilicity of membranes, blending allows lignin to change the mechanical properties of the membrane. The incorporation of nanoparticles as a filler at low loading levels can improve the immobilization of polymer chains in the membrane matrix, thereby improving rigidity and dimensional stability.^280,281^ Farooq et al.^282^ combined 10% (w/w) colloidal Kraft LPs with CNFs to produce composite films with double the toughness of a pristine CNF film. These lignin–CNF composites have potential applications in food packaging, water purification, and biomedicine. Manjarrez Nevárez et al.^283^ could also observe mechanical reinforcement when blending raw and propionated Kraft, organosolv, and hydrolytic LNPs into cellulose triacetate (CTA) water filtration membranes. This reinforcement effect was found to be dependent upon the film-casting conditions (temperature and relative humidity), the type of raw lignin, the lignin nanoparticle size, and the functional group content. All of the lignins, propionated or not, enhanced Young’s modulus and tensile strength compared to pure CTA membranes. The most prominent mechanical properties were observed for the CTA membranes blended with raw hydrolytic lignin, which demonstrated a Young’s modulus of 2.98 GPa, a 8.91% elongation at break, and a 128 MPa tensile strength. Propionated LNPs possess more propyl functional groups and fewer hydroxyl functional groups, yielding higher hydrophobicity and affinity for CTA, which is expected to contribute to the formation of more stable CTA membranes. However, this study showed that propionation improved the mechanical properties in CTA membranes only when Kraft lignin was used. Propionated organosolv or hydrolytic lignins did not result in the same gain in mechanical strength. Lignin nanoparticles are a competitive option as a reinforcing filler material due to the abundance of lignocellulosic material from which they can be produced,^284^ their low cost, and their absence of toxicity.^281^

In contrast to blending nanomaterial into the membrane substrate, another option is to modify only the membrane surface by using a nanomaterial coating. Taghipour et al.^285^ and Shamaei et al.^52^ utilized industrial waste lignin in the form of hydrophilic SKL to improve the antifouling performance of PA TFC and PES UF membranes, respectively. While membrane technology is a viable option for treating oily wastewater, the efficacy of the process suffers unless the membrane is treated to reduce its propensity for oil fouling. Kraft lignin was deposited on the membrane surface using a layer-by-layer assembly process with lignin as the polyanion and PDDA as the polycation.^52^ The highest antifouling performance was obtained for membranes coated with three PDDA/SKL bilayers assembled using 2% (w/w) solutions of both polyelectrolytes (membrane M3). During trial runs using a synthetic oil emulsion as the feedwater, M3 demonstrated a much lower flux decline (23%) and a much higher flux recovery ratio (93.8%) compared to that of the control pristine PES membranes (44.2% flux decline and 75.9% flux recovery). This could be attributed to the much higher surface hydrophilicity observed for M3 (22.6 ± 0.5° water contact angle) in comparison to that of the control PES membrane (71.1 ± 0.7° water contact angle). Functionalization with PDDA and Kraft lignin also decreased the molecular weight cutoff of the membranes from 19 kDa for pristine PES to 2–4.8 kDa depending on the number of layers applied during the layer-by-layer assembly, thereby improving membrane solute rejection. The trade-off for improved solute rejection is often decreased water permeability,^286^ which was also observed by Shamaei et al.^52^ Pure water flux decreased from 3.80 L/m^2^·h·psi for the pristine PES membrane to 1.6 L/m^2^·h·psi for M3. The lignin–PDDA coating on the surface of the PES UF membrane could be acting as a cake layer, which has been shown to function as a prefilter, decreasing UF membrane permeability and increasing selectivity.^287^ This is part of the downside of coating versus blending strategies for semipermeable membranes.

This work from Shamaei and co-workers^52^ helps to establish that Kraft lignin can impart organic fouling resistance to membranes in the form of oil fouling resistance. Improving membrane characteristics that contribute to organic fouling resistance, such as smoothness and hydrophilicity, can also increase the membrane’s resistance to bacterial adhesion and, thus, biofouling. Bergamasco et al.^288^ also observed an improved wettability (hydrophilicity) after coating electrospun PCL fiber membranes with a layer of acidolysis lignin. In another study, a lignin-based membrane layer is constructed on top of a PS support.^289^ This process is accomplished by using alkaline lignin and sodium LS as raw materials and dopamine (DA) as a surface modifier via brush-assisted layer-by-layer assembly. The resulting lignin-modified membranes demonstrate practical benefits such as ultrahigh water permeability (65.2 L/m^2^·h·bar), 100% MB rejection, and 85.1% NaCl permeation. The incorporation of lignin-based modification notably enhances fouling and chlorine resistance. For example, the lignin-modified membrane exhibits low flux decline and excellent flux recoverability during a 3-cycle Congo red fouling process despite its vulnerability to protein fouling.^289^ Karami et al.^290^ reported comparable findings for polyester membranes produced via interfacial polymerization of terephthaloyl chloride and SKL. These membranes exhibited a high negative surface charge, which contributed to their improved rejection performance against negatively charged dyes and certain organic foulants, such as bovine serum albumin (BSA).

Different approaches, rather than embedding or modifying the surface of MF and UF membranes with lignin, have been used to produce lignin-based water-filtrating materials. For example, researchers have used lignin and silk fibroin (SF) to create a multilayer nanofiber membrane using a layer-by-layer electrostatic spinning technique.^291^ They tested lignin derived from eucalyptus, pine, and wheat straw, and eucalyptus lignin was chosen due to its high content of hydroxyl groups and improved antibacterial properties. The eucalyptus lignin/SF membrane showed a superior retention rate for crystal violet, MB, and brilliant green dyes as well as metals such as cadmium and copper. Lignin played an important role in the chemical adsorption of the dyes through π–π conjugation and hydrogen bonding as well as electrostatic interaction with the cations. Another approach consisted of electrospinning sulfonated Kraft lignin (SKL), an industrial waste derivative of lignin, to build a nanofibrous support layer membrane^292^ or applying a copper-demethylated lignin interlayer between the substrate and selective layer to augment water permeability while maintaining salt rejection capabilities.^293^

### 5.2 Functionalization of the Polyamide Active Layer of Thin-Film Composite (TFC) Membranes with Lignin-Based Materials and Nanomaterials

In contrast to the porous membranes applied for MF and UF processes, the TFC membranes often employed for NF and RO are nonporous and impermeable under standard temperature and pressure conditions. TFC membranes do not function by mechanical sieving, instead they operate through a solution–diffusion mechanism,^294^ and high pressure must be applied to force water through the membrane. As previously mentioned, organic fouling and biofouling are of particular concern for NF and RO processes due to the composition of the TFC membranes. The gold standard for TFC membranes is a layer of PES covered in a selective layer of polyamide typically deposited by phase inversion.^286^ The polyamide active layer of TFC membranes is susceptible to oxidation, and for this reason, chlorine cannot be used to treat the feedwater as a preventative for biofouling. Instead, strategies for mitigating membrane fouling of TFC membranes should focus on preventing organic compound adsorption and bacterial adhesion with a minimal impact on the intrinsic transport properties.

Like semipermeable membranes, coating and blending techniques are often applied to modify TFC membranes. For TFC membranes, the coating method may be preferable for avoiding changes in the intrinsic transport properties of the TFC membrane. Zhang et al.^295^ utilized a coating of alkaline lignin to enhance the permeability and fouling resistance of RO filtration membranes without sacrificing selectivity. Lignin was deposited on the membrane surface through a simple filtration process that can be easily scaled up to commercial membrane modules. Filtering was used to apply the lignin coating to the membrane surface, relying on only hydrogen bonding and π–π interactions to maintain the lignin on the membrane surface. Despite this, the lignin coating was stable under cross-flow conditions and remained intact even after soaking of the membrane in deionized water for 3 months. While this method can be easily scaled up for commercial TFC membrane modules, the utilization of a method involving chemical bonding of the lignin to the membrane surface would likely improve the longevity and stability of the coating and should be further explored. No significant difference in hydrophilicity was observed for the coated and pristine membranes. Still, modified membranes did demonstrate slightly improved fouling resistance to both lysozymes (28% flux decline and ∼80% flux recovery) compared to the pristine TFC membrane (31% flux decline and ∼70% flux recovery). Additionally, the lignin coating improved the membrane water permeability and salt rejection, as well. The membrane coated using a suspension of 0.5 g/L lignin demonstrated a water permeability of 85.93 ± 0.61 L/m^2^·h, which was 24.2% higher than that of the pristine membrane (69.18 ± 0.13 L/m^2^·h). This same membrane demonstrated the highest salt rejection at 98.80 ± 0.24%, which was slightly higher than that of the pristine membrane (98.02 ± 0.09%).

In another recent study, lignin and aminated lignin (lignin–NH_2_) were used to create a thin-film layer through interfacial polymerization on the surface of an UF PES membrane to improve its hydrophilicity and antifouling properties.^273^ The incorporation of lignin–NH_2_ in the selective layer led to the enlargement of the pore size, increasing hydrophilicity, and improved negative charge. The lignin–PES membrane presented superior antifouling properties compared to its unmodified counterpart. The normalized flux of the lignin–NF membrane decreases to approximately 0.75, 0.8, and 0.65 in BSA, HA, and SA solutions, respectively. On the other hand, the normalized flux of the NF membrane decreases to around 0.5, 0.6, and 0.3 in the same solutions. This suggests that the lignin–NF membranes have better antifouling performance compared with NF membranes when exposed to all three types of foulants (Figure 6).

These findings are further supported by work conducted by Cusola et al.^296^ in which self-standing mold-casted membranes prepared from up to 92% Kraft LPs and CNFs resisted lysozyme adsorption. These LP-based membranes resisted disintegration in water, blocked the transmittance of UV light, and were found to be highly suitable for antioxidant MF. When compared to MF membranes prepared from solely CNFs, the LP-based membranes demonstrated an antioxidant capacity that was roughly 1000 times higher (824 ± 25 μmol of Trolox/mg) than that of the single-component CNF membranes (0.9 ± 0.08 μmol of Trolox/mg). Likewise, Chen et al. sought to prepare lignin-based membranes used for pervaporation desalination where lignin was the primary membrane substrate.^297^ High loadings of a commercial dealkalined lignin (ranging from 70 to 90%, w/w) were blended with just enough PVA to act as a binder and cast over a porous polyacrylonitrile support layer to form the TFC membrane. For the membrane composed of 90% lignin, the observed permeability (4.7 ± 0.16 kg/h·m^2^) and salt rejection (99.95%) were on par with the levels expected for TFC membranes. Their findings further demonstrate the potential for lignin as a next-generation membrane substrate.

Similar to semipermeable polymeric membranes, TFC membranes are also subject to the selectivity–permeability trade-off.^286^ Thus, there is a demand for strategies that can enhance the membrane permeability without the sacrifice of solute retention. Improving the membrane surface hydrophilicity could yield increases in the membrane performance in the form of permeability by increasing the surface wettability.^294^ The hydration layer formed on hydrophilic surfaces could allow for greater diffusion of water through the polyamide active layer of the TFC membranes. Shamaei et al.^298^ blended SKL into the selective layer of forward osmosis (FO) membranes to prepare antifouling composite membranes for the treatment of steam-assisted gravity-drainage-produced water. The membrane prepared with 6% SKL in the active layer demonstrated a water flux of 33.5 L/m^2^·h, nearly twice that of the pristine membrane when tested with 2 M NaCl as the draw solution. This was also attributed to increased hydrophilicity and negative surface charge caused by blending lignin into the membrane substrate before casting. This is demonstrated by the lower water contact angle observed for the M3 membrane (70.6°) in comparison to that of the control pristine membrane (88.7°). In a subsequent study, Shamaei and coauthors^299^ used SKL again to modify the selective layer of TFC FO membranes with the goal of producing antifouling membranes. Different concentrations (1, 3, and 6%, w/w) were blended into the polymerization reaction to form a SKL-containing selective layer. The membrane modified with 6% SKL presented a 2-fold improvement in water flux and lower flux decline under exposure to alginate solutions and synthetic wastewater compared to the unmodified membrane. For example, the pristine membrane presents a total flux decline ratio of 18.9%, while modified membranes with a maximum concentration of 6% (w/w) show only an 11.5% decrease. The antifouling properties relate to an increase in the wettability of the modified membranes after the incorporation of SKL. Similarly, Vilakati et al.^300^ also aimed to improve permeability through TFC FO membranes by increasing the membrane porosity through the inclusion of lignin in the membrane substrate.

Nanoparticles produced using sodium LS have been incorporated into the selective layer of TFC membranes.^301^ A mixture of spherical and elongated sodium LS particles was produced by the wet milling of lignin in hexane, resulting in average size distributions of 408 nm after 3 h and 234 nm after 5 h. The lignin particles were incorporated into the selective layer of PS membranes through spray-assisted interfacial polymerization. The modified membranes reduced the contact angle as water flux and salt rejection increased. Such an improvement in the membrane performance is due to an increase in the surface hydrophilicity and a more negative surface charge. Lignin has also been transformed into carbon dots (CDs) through hydrothermal processes and used to modify the surface of RO TFC membranes to impart antimicrobial properties^17^ (Figure 6). The CD-modified membrane effectively inhibited the viability of the attached cells of *E. coli* and *Bacillus subtilis* under exposure to UV and simulated daylight, suggesting that CD significantly improved the membrane’s ability to fight biofouling. For instance, TFC membranes modified with 500 μg/mL CDs inactivated 99.92 ± 0.03% of the attached *E. coli* cells compared to the TFC control membrane under UV light. This bacterial toxicity decreases when the CD membrane is tested in simulated wastewater, highlighting the need to optimize the CD coating to maximize antibacterial activity under realistic conditions. Although a mechanism of action was not demonstrated, the authors hypothesized that the CD particles can produce ROS in the presence of light (daylight or UV) and are probably deactivating bacteria cells through oxidation.

Despite lignin having the added benefit of being sustainable with abundant sources of renewable materials in the form of lignin-rich agroindustrial wastes, along with its low cost, nontoxic nature, and abundance of oxygen-containing groups, few studies have explored the use of lignin nanostructures for applications in membrane filtration processes. For example, lignin nanoparticles show morphological and surface chemistry characteristics similar to those of silica nanoparticles, the capabilities of which have been previously demonstrated by targeting this specific environmental application.^302,303^ This inference suggests that comparable results can be achieved from the lignin nanoparticles. The results outlined previously speak to the potential for valorization of both bulk lignin and lignin nanoparticles alike. However, further investigation is needed to understand the resulting characteristics of the lignin-modified membranes and the mechanisms leading to their antifouling and antibiofouling properties.

## 6 Lignin as a Potential Strategy in Antimicrobial Composites for Water Treatment and Remediation

The antimicrobial properties of lignin are closely related to its polyphenolic, heterogeneous structure. The chemical structure and composition of lignin can differ significantly depending on the taxonomy and maturation stage of the plant source, cell type, and environmental factors.^304,305^

**Figure 7:**
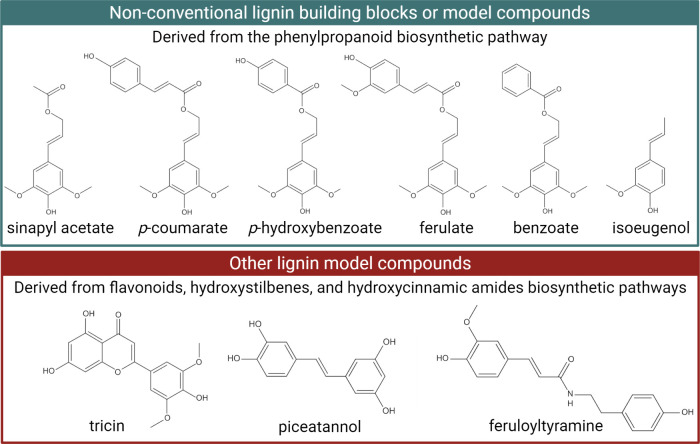
Chemical structures of nonconventional lignin building blocks (sinapyl acetate, *p*-coumarate, *p*-hydroxybenzoate, ferulate, and benzoate) or lignin model compounds (isoeugenol) derived from the phenylpropanoid/monolignol biosynthetic pathway and other lignin (polyphenolic) model compounds from beyond the canonical monolignol biosynthetic pathway, namely, flavonoid (tricin), hydroxystilbene (piceatannol), and hydroxycinnamamide (feruloyltyramine) biosynthetic pathways. Created in BioRender. Camargos, C. (2025) https://BioRender.com/i66o217.

Softwood, hardwood, and grasses vary in their composition of canonical monolignol precursors (Figure 2). Softwood and hardwood have higher proportions of guaiacyl and syringyl structural units, respectively.^28,305^ Minor concentrations of *p*-hydroxyphenyl building blocks are also present in grasses.^305^ Many other phenolic functional groups arising from the general phenylpropanoid biosynthetic pathway participate in radical coupling processes and are integrated into the lignin macromolecular network.^306^ Some examples include monolignol ester conjugates, which are present in various plants and can be found as acetates, *p*-coumarates, *p*-hydroxybenzoates, or ferulate and benzoate analogues (Figure 7).^307^ Other phenolic compounds, such as flavonoids, hydroxystilbene, or hydroxycinnamamide (Figure 7) originate from sources other than the conventional monolignol biosynthetic pathway.^307^ For instance, it has previously been observed that tricin, a flavone, is an important chemical constituent of lignin derived from many types of grass^308^ but not observed in gymnosperm lignins.^307−309^ Tricin can confer antimicrobial and antioxidant properties to the plant.^307,308^ Consequently, these differences in the lignin composition and structure may influence its antimicrobial properties.

### 6.1 Antimicrobial Effects of Lignin Model Compounds

The antimicrobial effect of model components of lignin has been studied since 1979 when low-molecular-weight fragments of lignin were tested against bacteria and fungi (*Saccharomyces cerevisiae*, *Candida albicans*, *E. coli*, *Bacillus licheniformis*, *Micrococcus luteus*, and *Aspergillus niger*).^310^ In this study, isoeugenol (Figure 7), which contains a methyl group at position γ and double bonds at positions α and β of the side chain, was found to be the most toxic fragment against all microorganisms, except for *S. cerevisiae*. Isoeugenol minimum inhibitory concentration (MIC) values ranged from 50 μg/mL (for *B. licheniformis* and *M. luteus*) to 100 μg/mL (for *C. albicans* and *E. coli*) to 250 μg/mL (*A. niger*). A MIC of 90 μg/mL was achieved using dehydrodiisoeugenol against *S. cerevisiae*.^310^ As a result, the side-chain structure of isoeugenol greatly influenced its antimicrobial properties.

In a more recent study, Yuan and colleagues^311^ found that the antibacterial properties of flavonoids were not determined by their unique chemical structure but rather by their polarity or lipid–water partition coefficients. The researchers explored the relationship between the physicochemical properties of plant flavonoids and their MICs against Gram-positive bacteria.^311^ Most flavonoids showed MICs ranging from 10.2 to 4.8 μM against Gram-positive bacteria, such as *S. aureus* and *B. subtilis*. The toxicity mechanism of plant flavonoids involves damaging the cell membrane, inhibiting the production of nucleic acids, suppressing bacterial respiratory chains, and destroying the cell envelope.^309,312^ Their antibacterial properties are strongly linked to their lipophilicity.^311^ Moreover, no clear relationship has been found between their toxicity and the chemical structure of their fragments. Such flavonoids are more likely to act nonspecifically on the cell–membrane bilayer or the respiratory chain.^311^

### 6.2 Antimicrobial Effects of Extracted and Technical Lignins

Apart from the biomass source, the extraction method is another essential factor that can influence the chemical structure and antimicrobial activity of lignin.^313,314^ It can have an impact on various characteristics of lignin, such as the size of the chains and the functional groups of phenolic fragments in the side chain, which, in turn, will determine the valorization route for converting lignin into value-added products.^313^

Lignins obtained through Kraft and sulfite processes have a high sulfur content varying from 3 to 8%.^62^ Conversely, soda and organosolv lignins are usually sulfur-free. Therefore, soda lignin can be used directly without purification and is suitable for chemical modification, allowing its utilization in cost-effective applications, including the manufacture of phenolic resins and dispersants.^315^ Additionally, the solubility of bulk lignins is also dependent on the extraction method. For instance, LS is a water-soluble form of lignin with high polydispersity and a broad molecular weight distribution.^62^

Examples of how the biomass source and extraction process combined can influence the antimicrobial activity of lignins are exemplified in the following studies. Composites made of hydroxypropyl methylcellulose (HPMC) and organosolv lignin extracted from softwood have shown improved antimicrobial activities compared to composites containing lignin produced from softwood through the Kraft process or organosolv lignin from grass.^314^ HPMC films containing organosolv lignin from softwood presented 6–7 log reduction against *S. aureus* in all lignin concentrations tested (5, 10, 15, 20, 25, and 30%, w/w), suggesting that *S. aureus* cells were sensitive to even low concentrations of organosolv lignin. In addition, these HPMC films containing organosolv lignin reduced the population of *E. coli* by 7 log cycles. On the other hand, HPMC films containing Kraft lignin exhibited the same log reduction for *S. aureus* at lignin concentrations of 20, 25, and 30% (w/w). The same HPMC films containing Kraft lignin only inactivated *E. coli* cells (6 log reduction) when lignin was present at a concentration of 30% (w/w). As the concentration of lignin in the film increased, a greater number of functional groups (aliphatic OH, carbonyl CO, and COOH) were incorporated into the film structure, which may be associated with the increasing antimicrobial activity of the fabricated films.^314^

Different extraction conditions, such as temperature and biomass/solvent ratio, were also found to affect the antimicrobial activities of lignin extracts. A study conducted by Dong et al.^316^ showed that alkaline lignin extracted from corn stover showed preferential antimicrobial activity to Gram-positive microorganisms. The extracts exhibited antimicrobial properties against *Listeria monocytogenes* and *S. aureus*, as well as yeast (*Candida lipolytica*). However, the lignin extract did not show toxicity to Gram-negative bacteria (*E. coli* O157:H7 and *Salmonella enteritidis*) or bacteriophage MS2. Extracts of lignin showed MIC values above 10 mg/mL against *L. monocytogenes* and the lowest MIC value for *S. aureus* (in the range of 1.25–5.625 mg/mL) when tested using a liquid incubation method in culture media.^316^ Similar patterns were also reported for soda and Kraft lignin extracts obtained from the liquor of different pulping conditions of bagasse and cotton stalks.^317^ These extracts were not toxic to Gram-negative bacteria (*E. coli*) and filamentous fungi (*A. niger*) but were effective against Gram-positive bacteria (*B. subtilis* and *Bacillus mycoides*). The highest antibacterial activity against these Gram-positive bacteria was seen for lignin produced from bagasse treated with soda and Kraft at 130 °C. No antimicrobial activity was identified after the cooking temperature was raised to 160 °C for 30 or 60 min.^317^

Although the precise antimicrobial mechanism of lignin has not been elucidated, these findings suggest that lignin is likely interacting and disrupting the integrity of the peptidoglycan layer of Gram-positive bacteria.^62,316,318^ However, this proposed mechanism must be approached with caution because previous studies have also shown toxicity against Gram-negative bacteria and fungi,^314,319^ indicating that the mode of action likely does not rely solely on the composition of the cell wall. The antimicrobial activity of lignin may also be attributed to interactions between its polyphenol functional groups and the surface properties of bacterial cells.

Quantitative structure–activity relationship models were developed to determine whether surface interactions between the polyphenol groups and components of the cell wall influence the antibacterial activity of lignin.^320^ A total of 35 phenolic compounds were tested at a 1 g/L against six bacterial strains individually: *S. aureus* CNRZ3, *B. subtilis* ATCC6633, *L. monocytogenes* ATCC19115, *E. coli* ATCC25922, *Pseudomonas aeruginosa* ATCC27853, and *S. enteritidis* E0220. They further evaluated the bacterial surface properties by microbial adhesion to solvents. Polyphenols exhibited a wide range of antibacterial activity against the tested strains, and it was unclear how specifically one class of polyphenols was related to the antibacterial activity. Among all compounds tested, a pattern was observed in which the same polyphenol was found to be toxic to one Gram-positive or Gram-negative strain but ineffective toward others, showing that its antibacterial property is strain-dependent. This heterogeneity in toxicity may be due to the surface properties of the bacterial cell wall. Gram-positive *S. aureus* and *L. monocytogenes* have an amphiphilic surface, while *P. aeruginosa* and *B. subtilis* possess a polar surface. The cell walls of *E. coli* and *S. enteritidis* demonstrated an intermediate polarity between the previous two groups. Thus, the primary mechanism of action would rely on the adhesion of polyphenols to the cell wall of bacteria cells, a process facilitated by the hydrophobic character of the polyphenols and the surface properties of these bacteria.

To optimize the use of lignin as an antimicrobial agent and tailor its properties to develop advanced functional materials, it is essential to consider the extraction method and the target microorganism. For example, combining organosolv and Kraft methods rendered a low-molecular-weight lignin (*M*_w_ = 1810 g/mol) with a higher content of phenolic functional groups, which was toxic to *E. coli* (inhibition ratio of 95.61%), *Salmonella enterica* subsp. *enterica* serovar Typhimurium (89.60%), *Streptococcus pyogenes* (66.62%), and *S. aureus* (64.68%).^318^ The reported toxicity has been attributed to the pH lowering around the cell membrane caused by the phenolic groups.

### 6.3 LNP-Based Antimicrobial Composites

In addition to bulk lignin, lignin-based nanomaterials have attracted attention due to their unique physical and chemical characteristics, which are associated with their small size and high surface area.^49,321^ LNPs have been used to produce polymeric composites with enhanced UV shielding,^27,322^ antioxidant,^323,324^ and antimicrobial properties.^324−326^

There has been an increased interest in the toxicity of LNPs to bacteria cells. For example, LNPs produced by the dissolution of pristine alkaline lignin into ethylene glycol under different acidic conditions were found to be effective antibacterial agents to phytopatogenic Gram-negative bacteria, including *Pseudomonas syringae* pv *tomato* (CFBP 1323, *Xanthomonas axonopodis* pv *vesicatoria* (CFBP 3274), and *Xanthomonas arboricola* pv *pruni* (CFBP 3894).^327^ A LNPs suspension prepared using 2.5 M HCl from alkaline lignin in ethylene glycol produced LNPs with the highest yield (87.9%), smallest size (32 nm), and better thermal stability (18.6% residual mass at 800 °C). Three different assays (spot diffusion, incorporation, and growth in media broth) were used to assess the antibacterial activity of the LNPs against plant pathogens. By the application of spot diffusion assay, *P. syringae* was the most susceptible bacteria to the LNPs at concentrations of 5% and 8% (w/w). After 24 h of growth in broth with 4% LNPs, *X. arboricola* showed a 3 log decrease in cell viability. A total inhibition in growth was obtained for all bacterial strains when 4% LNPs were incorporated into the growth medium.

LNPs are also combined with polymers or other nanomaterials to produce lignin-based antimicrobial composites. For instance, ternary poly(l-lactide) (PLLA)/LNP-based materials showed a more pronounced antibacterial activity against *S. aureus* than against *E. coli* in general.^324^ Among all nanocomposite films tested, the highest values of antibacterial activity were shown by PLLA/1LNP/0.5Ag_2_O (60%) against *S. aureus* and by PLLA/1LNP/0.5ZnFe_2_O_4_ (35%) against *E. coli* at 6 h. The assays were performed on the planktonic bacterial cultures removed after being in contact with PLLA and PLLA nanocomposite films containing LNPs and/or metal oxide nanoparticles for 6 and 24 h at 37 °C. The same pattern has been found previously, with bulk lignins presenting higher antimicrobial activity against Gram-positive bacteria in contrast to Gram-negative bacteria.

The mechanism of action causing the toxicity of LNPs to bacteria cells is not yet well understood because there are only a few studies on the topic. Slavin and colleagues^328^ were the first to publish a comprehensive study that included the characterization of physical and chemical properties, antimicrobial activity, and the impact on bacterial gene expression of silver-LNPs (AgLNPs) hybrid materials. To produce silver nanoparticles (AgNPs) in an environmentally friendly way, the authors utilized lignin (alkali-low sulfonate) as a reducing and capping agent. The resulting AgLNPs hybrid material was tested against a panel of Gram-positive and Gram-negative multidrug-resistant (MDR) clinical bacterial isolates, including *S. aureus*, *Staphylococcus epidermidis*, *P. aeruginosa*, *Klebsiella pneumoniae*, and *Acinetobacter baumannii*, and a variety of American Type Culture Collection (ATCC) bacterial strains. AgLNPs showed MIC values between 5 and 25 μg/mL while appearing to be more toxic to MDR than ATCC strains. MICs for ATCC strains reached up to 25 g/mL. When tested up to 50 g/mL, bulk lignin on its own had no antibacterial effect, and AgNPs without lignin required a 5-fold higher concentration to achieve the same performance as AgLNPs.

Lignin appears to change the stability and compactness of the model bacterial membrane, allowing soluble Ag^+^ ions to penetrate the bacterial cells and result in increased antibacterial activity. *K. pneumoniae*, *S. aureus*, and *P. aeruginosa* internalized the AgLNPs, while *A. baumannii* experienced adsorption of the AgLNPs. The study also examined how exposure to AgLNPs at a sublethal concentration of 2.5 μg/mL affected the gene expression of *P. aeruginosa*. The research focused on genes involved in regulating cell membrane efflux, heavy-metal resistance, capsular biosynthesis, and quorum sensing. The results showed that genes responsible for membrane proteins with an efflux function were upregulated (increased), while all other genes encoding membrane proteins that did not efflux metals were downregulated (suppressed).^328^

The antimicrobial properties of LNPs may be due to their photodynamic activity.^329,330^ When exposed to light, LNPs can produce ROS, which leads to the oxidation and peroxidation of membrane lipids, causing the membranes to rupture and the cells to leak. Paul et al.^329^ introduced a one-step green method to develop a lignin-nanosphere-based spray (LNSR) and evaluated its antimicrobial photodynamic therapeutic activity against *E. coli* and *Bacillus megaterium* (Gram-negative and Gram-positive bacteria, respectively). The results showed that LNSR had a stronger antimicrobial effect against *E. coli* when irradiated with a blue LED (12 W), with a half-maximal inhibitory concentration (IC50) of 74.40 mg/mL. In contrast, the IC50 almost doubled to 137.12 mg/mL in the dark. For *B. megaterium*, the IC50 was 77.68 mg/mL under UV light and 114.93 mg/mL in the dark. When treated with LNSR and exposed to light, cells exhibited a higher genetic material leakage, and surface charge studies confirmed membrane rupture. Cells treated with LNSR under light exposure had significantly lower surface charge compared to untreated cells.

Tanganini and collaborators^15^ evaluated the production of ROS by LNPs through in vitro and in vivo assays and showed that oxidative stress was not the primary mechanism of antimicrobial action of self-assembled lignin nanoparticles (SA-LNPs). In this study, lignin derived from lignocellulosic biomass (elephant grass) was converted to antimicrobial nanoparticles using a straightforward self-assembly method. These SA-LNPs exhibited spherical morphology, an average size of 80 nm, and a surface charge of −29 ± 4 mV. The antibacterial efficacy of SA-LNPs was tested against four bacterial strains: *E. coli* and *P. aeruginosa* (Gram-negative); *B. subtilis* and *Lactobacillus fermentum* (Gram-positive). The results from antimicrobial assays in saline media demonstrated that SA-LNPs were selectively toxic to Gram-positive bacteria with no significant effects on Gram-negative strains. Time-kill experiments revealed that 25 μg/mL SA-LNPs inactivated over 90% of Gram-positive bacteria within 30 min of exposure. SA-LNPs were found to have strong antioxidant properties, as evidenced by their ability to scavenge the 2,2-diphenyl-1-(2,4,6-trinitrophenyl)hydrazin-1-yl free radical. Despite ruling out direct oxidative stress, the potential for an indirect pro-oxidant effect due to ROS adsorbed on SA-LNPs interacting with bacterial cell walls cannot be entirely dismissed.^15^

LNPs have also been used as organic matrixes to encapsulate photosensitizer molecules such as porphyrin (THPP) to enable antimicrobial activity through photodynamic processes.^330^ Acetylated LNPs (@AcLi) and THPP-loaded @AcLi (THPP@AcLi) were produced and tested against five bacterial strains: three Gram-positive (*S. aureus*, *S. epidermidis*, and *Enterococcus faecalis*) and two Gram-negative (*E. coli* and *P. aeruginosa*). After 1 h of irradiation with a white LED light dosage of 4.16 J/cm^2^, THPP@AcLi revealed a high ability to inhibit the growth of Gram-positive bacteria. Concentrations as low as 0.078 mM were sufficient to inactivate Gram-positive growth by approximately 85%. However, THPP@AcLi was unable to inactivate the Gram-negative *E. coli* but seemed to have a bacteriostatic nonphotodynamic effect on *P. aeruginosa*. Transmission electron microscopy images detected @AcLi on the surface of *S. aureus* cells. This finding indicates that @AcLi did not enter the cell but instead stayed on the perimeter of the cell, causing local ROS production surrounding the cell wall.^330^ It was also observed that the bacteria and @AcLi immediately flocculated to the bottom of the assay flask, suggesting a high affinity of the bacteria by THPP@AcLi.

In the context of lignin-based antimicrobial and antibiofouling coatings and composites for water treatment technologies, the most impactful studies have concentrated on developing membranes, as detailed in Section 5 of this review. The antimicrobial results previously described are paramount to understanding the role of lignin-based materials as antimicrobial agents and the mechanisms behind their toxicity, allowing for their effective application to inactivate or inhibit target microorganisms during water treatment and remediation processes.

**Figure 8:**
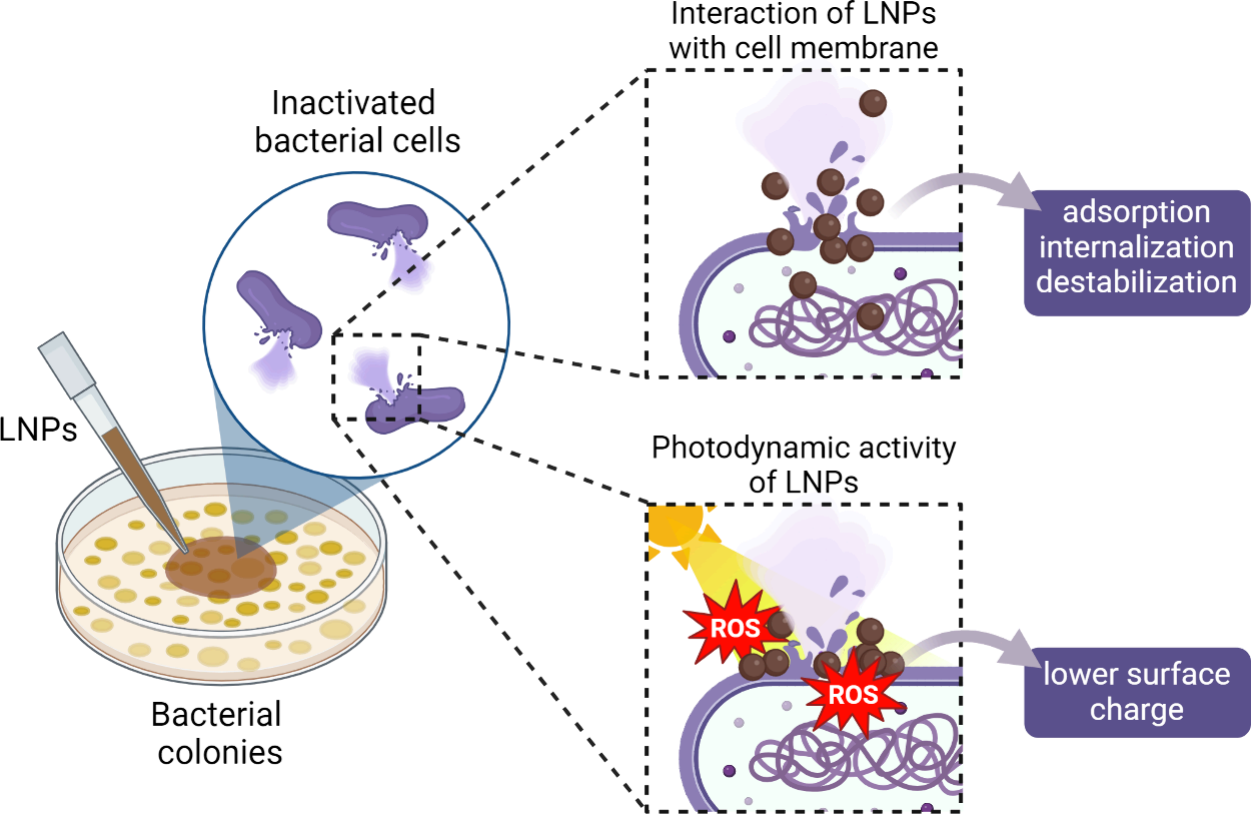
Schematic depiction of the two major antimicrobial mechanisms of LNPs, leading to lysis of the intracellular content. LNPs can interact with the cell membrane through adsorption, internalization, and destabilization. Alternatively, under photodynamic stimuli, LNPs can generate ROS locally, lowering the surface charge and causing significant damage, thereby disrupting the cell membrane. Created in BioRender. Camargos, C. (2025) https://BioRender.com/o90i747.

Figure 8 provides a summary of the two major mechanisms underlying the antimicrobial activity of LNPs. As mentioned, the antimicrobial effect of lignin-based materials varies depending on factors such as the type of biomass precursor and extraction method,^313,314^ the molecular weight of its macromolecular chain fragments,^327^ and its interactions with other components.^311^ The composition of the cell wall and surface charge also play a significant role in the toxicity of lignin-based materials to microorganisms.^15,320^ Although the exact mechanism of toxicity is not fully understood, the antimicrobial properties likely result from multiple processes occurring upon contact of cells with the LNPs. These processes may include destabilization of the bacteria cell wall,^331^ generation of ROS,^329^ and alteration of microbial signal transduction pathways.^328^

## 7 Economic Assessment of Lignin-Based (Nano)materials for Water Treatment

The economic evaluation of water treatment methods utilizing lignin-based adsorbents, photocatalysts, flocculants, membranes, and antimicrobials along with life cycle assessment is crucial for estimating the feasibility and scalability of these systems.

Operational expenses associated with lignin utilization vary based on the feedstock and extraction method. For wood-derived lignin, production costs range from $250 to $500 (US dollars) per ton (t) of KL, equivalent to $0.25 to $0.5 per kilogram (kg). The costs for LSs range from $300 to $2700/t or from $0.3 to $2.7/kg.^332^ Extraction costs for lignin from nonwood lignocellulosic biomasses, such as sugar cane bagasse and rice husk, are relatively higher, demanding $4.2–6.0/kg for soda extraction, $4.8–6.8/kg for the Kraft process, $7.2–10.7/kg for organosolv extraction, and $5.6–8.0/kg for LS extraction.^19^ A technoeconomic assessment by Abbati de Assis and co-workers^332^ estimated the manufacturing costs of wood-derived lignin micro- and nanoparticles to be between $871 and $1168/t (equivalent to $0.87–1.17/kg), with a minimum selling price ranging from $1241 to $1559/t ($1.24–1.56/kg). For comparison, AC produced from spent coffee grounds has a minimum selling price of $0.15–0.28/kg,^333^ while production costs for AC derived from oil palm waste fall between $2.72 and 3.24/kg.^334^ Furthermore, the production costs for various flocculants vary: PAC at $215/t ($0.22/kg),^335^ PAM at $167/kg, and aluminum sulfate at $77/kg.^336^

There is still limited literature that comprehensively addresses the capital and operating costs associated with using lignin-based nanomaterials for environmental remediation. In well-established scenarios, wastewater treatment plants that utilize traditional coagulants, such as aluminum sulfate, often incur high treatment costs (measured in $130/t) and generate large volumes of sludge, which lead to increased carbon emissions.^337^ For textile wastewater, the treatment costs with aluminum sulfate range from $0.28 to $0.75/m^3^, resulting in the production of up to 1.7 kg/m^3^ of sludge.^338^ In contrast, a flocculant based on lignin-based cationic polyelectrolyte reduced the sludge production to only 4.0–5.3% after dye removal.^246^ However, comprehensive global cost data for this alternative material are still lacking.

Using biobased materials can be advantageous for wastewater treatment plants because they generate less sludge, enhancing environmental sustainability and lowering the costs associated with secondary pollution management.^339^ A technoeconomic analysis by Gujjala and Won^340^ indicated that the minimum selling price for a lignin-based hydrogel fabricated by copolymerization of LS and acrylic acid ranges from $1965 to $2141/t ($1.96–2.14/kg). This price falls within the market range for commercial coagulants used to treat dye-contaminated wastewaters ($1420–2280/t or $1.42–2.28/kg). Although the economic implications of using lignin hydrogel were not discussed in this study, it was assumed that the cost of a lignin precursor (LS) would be 12.5 times lower than that of acrylic acid, which is priced at $2250/t.^340^

To compare conventional and lignin-based water treatment processes, we estimated costs based on lignin prices and average dosages of lignin-based adsorbents (macro-, micro-, and nanolignin). These calculations are approximations and may lack significant consistency because they only consider the maximum lignin production cost of $10.7/kg and dosages ranging from 0.05 to 1.0 g/L (Table 1). Additionally, we have not factored in costs related to energy, reuse and recyclability, other reagents, or economies of scale. Based on these assumptions, the estimated cost for wastewater treatment, using only pure lignin materials, would be approximately $0.54–10.7/m^3^.

Lignin-based water treatment technologies present considerable economic and environmental advantages, positioning them as compelling alternatives to conventional methods. Nonetheless, challenges related to cost and processing still exist, underscoring the necessity for advancements in scalability and process optimization to enhance efficiency and competitiveness. Furthermore, comprehensive economic feasibility studies and life cycle assessments are vital for the thorough evaluation of their scalability and long-term sustainability.

## 8 Challenges and Perspectives

In the midst of the pressing global challenge to ensure safe management of clean and potable water, lignin shows promise as a sustainable solution for water treatment technologies. However, several challenges must be tackled to fully harness its potential in cost-effective, efficient, and accessible applications.

Extraction of lignin from lignocellulosic biomass often involves complex processes such as acid hydrolysis, alkaline treatment, and Kraft, sulfite, or organosolv methods. Performing and scaling up efficient extraction while maintaining the structural integrity (or traceability) and purity of lignin poses a significant challenge.

The intricate and diverse nature of lignin presents several obstacles in establishing standard properties for a consistent performance in water treatment processes. The varied and heterogeneous structure of lignin molecules, which is affected by factors like the biomass source and extraction method, can influence its efficacy in adsorption, flocculation, photocatalysis, and antimicrobial treatments. It is crucial to comprehend and manage these structural differences to maximize the effectiveness of using lignin-based materials in water treatment.

The integration of lignin-based materials into the existing water treatment infrastructure may entail technical challenges. Compatibility issues could arise due to differences in material properties, such as the particle size, surface charge, and stability, which can affect the performance and longevity of filtration membranes, adsorbents, or flocculants. Adapting conventional treatment processes to accommodate lignin-based materials requires careful consideration of the operational parameters and system dynamics. However, it could also benefit largely from the abundance, low cost, versatility for chemical modification, and antioxidant properties of this polyphenolic macromolecule.

Similarly, utilizing lignin for water treatment applications has the advantage of being highly scalable because lignin-rich streams are byproducts of paper, bioethanol, and other agroindustrial processes. Nonetheless, extracting lignin may present economic challenges due to the use of energy-intensive processes and expensive chemicals. Developing cost-effective and sustainable extraction techniques is crucial to making lignin-based water treatment technologies commercially viable on a large scale.

It is also important to consider the potential environmental and health impacts of lignin-based strategies. Lignin is an aromatic compound derived from renewable sources and is considered nontoxic. However, its ecological and health effects need to be thoroughly evaluated. The use of lignin-based materials in water treatment processes could potentially introduce new pollutants or byproducts into the environment if not properly managed. Therefore, there is a need to assess the life cycle impacts of lignin (nano)material production and disposal to ensure the overall sustainability of lignin-based water treatment technologies.

Finally, to optimize the performance of lignin-based materials in water treatment applications, we need to understand better how they interact with contaminants under realistic environmental conditions. We can tailor lignin properties through chemical modification, nanoparticle engineering, or composite formulation to enhance its adsorption capacity, photocatalytic and antimicrobial activities, and flocculation efficiency. However, it is a great challenge to boost these parameters while keeping the process cost-effective and sustainable. Despite the existence of these obstacles, lignin-based materials and nanomaterials have the potential to transform water treatment technologies, providing sustainable and cost-effective solutions to address global water resource challenges.

## 9 Conclusions

To effectively address global water management challenges, it is essential to adopt innovative and sustainable strategies to ensure widespread access to clean drinking water. Lignin, an abundant and renewable byproduct of the cellulose and bioethanol industries, has emerged as a promising material for advanced water treatment technologies. This review underscores the versatility of lignin-based materials: ranging from macro- to micro- to nanostructures, in applications such as adsorption, catalysis, flocculation, membrane filtration, and antimicrobial functions, emphasizing their potential for scalability and cost-effectiveness. Challenges continue to persist in optimizing lignin extraction, standardizing its properties, and integrating it into existing water treatment systems, while ensuring both environmental and economic sustainability. By enhancing our understanding of the interactions between lignin and contaminants and by harnessing the multifunctional properties of this macromolecule, lignin-based materials can make a pivotal contribution to the development of effective, environmentally friendly, and accessible water treatment solutions. This advancement could be instrumental in working toward the achievement of Sustainable Development Goal 6 (SDG 6).

## Abbreviations

AC: activated carbon
AgNPs: silver nanoparticles
AL: alkaline lignin
AOP: advanced oxidation processes
BPA: bisphenol A
CD: carbon dot
CF: carbon nanofiber
CNF: cellulose nanofibril
CTA: cellulose triacetate
DMC: (methacryloyloxyethyl)trimethylammonium chloride
EHL: enzymatic hydrolysis lignin
FO: forward osmosis
g-C_3_N_4_: graphitic carbon nitride
GO: graphene oxide
HPMC: hydroxypropyl methylcellulose
KL: Kraft lignin
LC: lignin-based carbon
LNP: lignin nanoparticle
LP: lignin particle
LS: lignosulfonate
MB: methylene blue
METAC: [(2-methacryloyloxy)ethyl]trimethylammonium chloride
MF: microfiltration
MG: malachite green
MIC: minimum inhibitory concentration
MO: methyl orange
NF: nanofiltration
PAC: poly(aluminum chloride)
PAM: polyacrylamide
PCL: polycaprolactone
PDDA: poly(diallyldimethylammonium chloride)
PLLA: poly(l-lactide)
PES: poly(ether sulfone)
PVC: poly(vinyl chloride)
RhB: Rhodamine B
RO: reverse osmosis
ROS: reactive oxygen species
RY4G: reactive yellow 4G
SKL: sulfonated Kraft lignin
SMX: sulfamethoxazole
TFC: thin-film composite
THF: tetrahydrofuran
TMP: trimethoprim
TNT: 2,4,6-trinitrotoluene
TOC: total organic carbon
UF: ultrafiltration
